# Recovery of lithium from a zinnwaldite-bearing concentrate derived from tailings using commonly available flue-gas desulfurization gypsum

**DOI:** 10.1039/d6ra04143d

**Published:** 2026-07-03

**Authors:** Martin Šulc, Ladislav Bříza, Jiří Orava, Pavel Janoš

**Affiliations:** a Faculty of Environment, J. E. Purkyne University in Usti nad Labem Pasteurova 3632/15 Ústí nad Labem 40096 Czech Republic pavel.janos@ujep.cz

## Abstract

Natural gypsum and flue-gas desulfurization (FGD) gypsum were investigated as sulfate additives for lithium recovery from a zinnwaldite-bearing concentrate obtained from the Cinovec tailings repository. The performance of the gypsum materials during calcination followed by water leaching was compared with that of laboratory-grade calcium sulfate at an equivalent calcium dosage. For all additives, the principal operating window was centred at approximately 950 °C with an annealing time of 60–90 min, where high lithium recoveries of *ca.* 93–96% were obtained. Raising the temperature to 1000 °C did not improve recovery and, in some cases, even decreased it. Relative to natural gypsum, FGD gypsum showed a more homogeneous morphology, higher Ca and S contents, and lower levels of silicate impurities. Nevertheless, the differences in process performance between the two industrial gypsum sources were modest. Milling the concentrate prior to calcination increased lithium recovery from 81.7% to 98.6%, whereas intensive milling of the calcined clinker decreased the apparent recovery from 97.8% to 87.2%, most likely because ultrafine particles hindered filtration and retained Li-bearing solution. These results indicate that FGD gypsum is a practical alternative to reagent-grade calcium sulfate for gypsum-assisted lithium recovery from zinnwaldite. More importantly, the comparable performance of FGD gypsum, natural gypsum and laboratory-grade CaSO_4_ shows that the gypsum-assisted route is not restricted to model reagents but can tolerate technically realistic sulfate sources. Beyond demonstrating the practical impact, this study elucidates the complex solid-state reaction pathways and kinetic boundaries that govern lithium mobilization when utilizing industrial by-products. By mapping these phase transformations, this work advances the fundamental chemical understanding necessary to recover critical materials from secondary waste streams.

## Abstract 2

From lithium waste to Li product *via* sulfate roasting, efficient use of gypsum by-products.

## Graphical abstract

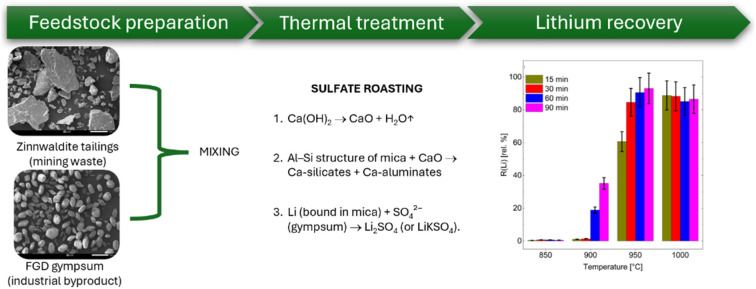

## 1. Introduction

With the development of lithium-ion batteries and their crucial role in e-mobility and stationary energy storage, interest in lithium has shifted from a geochemical curiosity to a geopolitical commodity playing a dominant role in the considerations and decisions of politicians around the world.^1–3^ Current lithium production relies on both primary and secondary resources and increasingly emphasizes technological routes that aim to reduce chemical consumption, waste generation, and environmental burdens.^2,3^ Brines and spodumene (∼7 wt% of Li_2_O) concentrates currently drive the industrial production of lithium, in the form of LiOH and Li_2_CO_3_.

A recent comprehensive review has summarized the current landscape of lithium extraction from both hard-rock and brine resources, including conventional pyrometallurgical and hydrometallurgical routes, as well as emerging direct lithium extraction technologies.^4^ The strategic importance of alternative and non-traditional hard-rock resources, for example amblygonite, lepidolite, petalite or swinefordite, has increased, particularly in regions aiming for domestic supply diversification and enhanced resilience of critical mineral supply chains.^1–3^

Among hard-rock alternatives, lithium micas occupy a particular niche source and are commonly discussed in the context of rare-metal granite and greisen districts associated with Sn–W mineralization.^5–7^ However, lithium in micas is strongly lattice-bound within a complex aluminosilicate framework, and its extraction typically requires a disruptive pre-treatment (thermal, chemical, or mechanochemical) followed by leaching and downstream purification.^8–10^ Typical routes for obtaining lithium from micas include beneficiation and upgrading, acid or alkaline leaching, and roasting-based activation routes such as sulfate, chloride/chlorination, or alkaline salt roasting, highlighting that mineralogy, gangue assemblage and impurity suite largely dictate the optimum flowsheet.^8–10^ Microwave-assisted leaching was recently suggested to intensify the extraction process.^11^

Zinnwaldite is an iron-rich lithium mica with the general formula KLiFeAl(AlSi_3_)O_10_(OH,F)_2_, found in greisen and rare-metal granite systems. It occurs in the Cinovec/Zinnwald region, where historical and current activities have generated mica-rich waste streams suitable for upgrading.^5–7^ The Cinovec/Zinnwald deposit is regarded as one of the most significant lithium resources in Europe and is therefore relevant to ongoing EU efforts to strengthen the domestic supply of critical raw materials.^1,12^ A detail often overlooked outside mineralogical circles—yet it can be important technologically—is that “zinnwaldite” is usually regarded as a compositional term covering the polylithionite-siderophyllite series rather than a strictly fixed end-member.^13,14^ Consequently, the chemistry of zinnwaldite may vary, leading to different behavior and phase evolution upon heating and leaching. The management of iron- and fluorine-bearing components represents an important technological challenge, while fluorine may also pose an environmental concern, especially during thermal treatment.^13–15^ This complex mineralogical composition explains why activation conditions optimized for one “zinnwaldite” feed cannot be automatically transferred to other feeds.

To place zinnwaldite-type resources in a broader chemical and technological context, Table 1 summarizes representative lithium-bearing primary and secondary resources, including their approximate Li_2_O or Li_2_O-equivalent contents and the principal processing chemistry.

**Table 1:** Representative examples of lithium-bearing resources and their processing chemistry lithium contents are expressed as Li_2_O equivalents where applicable. For pure minerals, values are theoretical stoichiometric values; for ores, concentrates, brines and recycled streams, values are representative literature or process-relevant ranges^a^

Representative example/resource type	Main Li host or source	Typical Li_2_O or Li_2_O-equivalent content	Main advantage/limitation	Key processing chemistry
Spodumene pegmatites (*e.g.*, Greenbushes; hard-rock concentrates)	Spodumene, LiAlSi_2_O_6_	Theoretical mineral: 8.0 wt% Li_2_O; real mineral composition 2.9–7.6 wt% Li_2_O;^8^ in concentrates lower values, *e.g.* 7,2 wt% Li_2_O^4^	High grade and mature technology; energy-intensive decrepitation/roasting	α-spodumene → β-spodumene phase conversion; H_2_SO_4_ roasting to Li_2_SO_4_; water leaching and Li_2_CO_3_/LiOH production
European hard-rock mica resources (*e.g.*, lepidolite-rich pegmatites)	Lepidolite, K(Li,Al)_3_(Si,Al)_4_O_10_(OH,F)_2_ Li-bearing micas, mainly lepidolite series	Lepidolite 3.7 wt% Li_2_O;^4^ mica concentrates often *ca.* 1–4 wt% Li_2_O depending on beneficiation^8–10^	European relevance and possible beneficiation; lower grade and complex aluminosilicate matrix	Sulfate/chloride/alkaline roasting disrupts mica lattice; Li is converted to soluble sulfate, chloride or carbonate species before leaching
Cinovec/Zinnwald-type greisen and tailings	Zinnwaldite, KLiFeAl(AlSi_3_)O_10_(F,OH)_2_	Reported concentrate: 1.40 wt% Li, equivalent to *ca.* 3.0 wt% Li_2_O^5,6^	Regionally relevant European resource; lower grade, compatible with CaSO_4_/Ca(OH)_2_ processing	Gypsum route: CaSO_4_/Ca(OH)_2_-assisted roasting decomposes mica and converts structural Li into water-leachable Li sulfate phases
Lithium-rich clays (*e.g.*, hectorite/illite-smectite deposits)	Li in clay interlayers and octahedral sheets	Site-specific; commonly lower than in hard-rock concentrates^4^	Large tonnage potential; slow leaching and difficult solid–liquid separation	Acid or alkaline leaching after activation; aluminosilicate/clay dissolution and selective Li recovery from impurity-rich liquors
Continental salar brines (*e.g.*, Atacama-type brines)	Dissolved Li^+^ in hypersaline brines	Site-specific, *ca.* 10^−3^–10^−1^ wt% Li^4^	Low mining intensity and established evaporation routes; water use, Mg/Li ratio and long residence times are limiting	Evaporation or direct lithium extraction; precipitation, ion-exchange or sorption chemistry; final conversion to Li_2_CO_3_ or LiOH.
Spent Li-ion battery black mass	Li in cathode phases, electrolyte residues and surface salts	Highly variable; comparable to hard-rock concentrate	High-value secondary resource; safety, fluorine, graphite and transition metals complicate processing	Discharge and pretreatment followed by hydrometallurgical leaching; pH/redox control separates Ni, Co, Mn, Fe, Al, Cu before Li purification

a Note: Li_2_O equivalent from Li is calculated as Li × M(Li_2_O)/(2M(Li)) = Li × 2.153; therefore 1.40 wt% Li corresponds to approximately 3.01 wt% Li_2_O.

Technological processes developed for extracting lithium from micas have primarily focused on activation strategies to mobilize lithium into water- or acid-soluble phases while leaving matrix constituents and unwanted impurities as insoluble products.^8–10^ Among these approaches, sulfation roasting has been studied particularly extensively because it can convert lithium into soluble sulfates or mixed alkali sulfates, while favoring the formation of relatively refractory Ca-(Al,Fe)-silicates in the residual slag.^8–10,16–19^ The origin and activity of sulfate sources can influence leaching kinetics and thermodynamic stability, thereby controlling overall lithium recovery.^10,13,18,19^ In addition, sulfate roasting in Ca-bearing systems may contribute to fluoride immobilization in sparingly soluble solid phases.^20^

Despite extensive laboratory studies on the roasting and leaching of lithium micas published in the literature.^5,6,21,22^ a limitation remains, as most studies rely on reagent-grade additives with well-controlled properties. By contrast, industrial sulfate sources such as natural and flue-gas desulfurization (FGD) gypsum may differ substantially from reagent-grade calcium sulfate in moisture content, hydration state, particle size and distribution, and impurity levels,^23,24^ all of which can significantly affect the complex processes involved in calcium sulfate-assisted lithium extraction from zinnwaldite. While the fundamental reaction stoichiometry of idealized sulfate roasting is well documented, the complex solid-state chemistry occurring within highly heterogeneous, multicomponent secondary resources remains of high interest. Utilizing industrial by-products like FGD gypsum introduces competing phase transformations and altered kinetic pathways. Therefore, this study aims not only to demonstrate technical feasibility but to fundamentally investigate the chemical reaction dynamics, phase evolution, and kinetic limitations—modeled *via* a Bateman-type function—that govern this complex waste utilization system.

In this work, natural and FGD gypsum are evaluated as technologically viable sulfate additives for the roasting of zinnwaldite-bearing feedstock and subsequent lithium recovery. The present study (i) compares the two gypsum sources under controlled Ca/S input and roasting conditions; (ii) establishes an operational temperature – time window that maximizes lithium recovery; and (iii) quantifies the influence of feed and clinker particle size on extraction performance. Overall, the results provide an industrially relevant basis for selecting gypsum-based additives and for designing a robust roasting – leaching route that can accommodate realistic variability in raw-material quality.^16–19,23^

## 2. Experimental

### 2.1. Materials and chemicals

Li concentrate was prepared from a bulk sample collected from the Cinovec tailings repository in the Czech Republic, which consists of waste material generated during historical Sn–W mining. Pre-concentration of the material was carried out by dry magnetic separation. A representative sample of the Li concentrate was obtained by homogenization of the entire material lot. Flue-gas desulfurization (FGD) gypsum was obtained from a wet limestone scrubbing unit at the Ledvice power plant (Czech Republic). Natural gypsum was collected from a surface gypsum quarry in Koberice near Opava (Czech Republic). Commercial calcium hydroxide (CL 90-S) was supplied by Lhoist and produced from limestone originating from the Certovy schody quarry (Tman, Czech Republic); the material complied with the technical requirements of CSN EN 459–1. Unless stated otherwise, all other chemicals used in the experiments were of laboratory grade and were purchased from Lach-Ner (Neratovice, Czech Republic).

Prior to use, all solid materials were stored in sealed plastic containers to avoid moisture uptake. Both gypsum materials were pre-dried at 160 °C for 4 h. The natural gypsum sample was additionally milled in a planetary ball mill (Fritsch Pulverisette 5) at 300 rpm for 10 min using six corundum balls (3 cm diameter) as the milling media with the goal of obtaining a comparable granulometry to the FGD gypsum.

More detailed characterization of the materials used in this work is given in the SI (SI), Chapter S1.

### 2.2. Calcination and leaching experiments

The calcination procedure used in this study was based on the original gypsum method for processing lithium micas^5,6^ and was modified to meet the objectives of the present investigation. The reference feed mixture was prepared from the Li concentrate, CaSO_4_ and Ca(OH)_2_ in a mass ratio of 6 : 4.2 : 2. For the experiments with natural and FGD gypsum, the mixture design was adjusted to maintain a constant calcium input across the investigated feed compositions. The effects of calcination temperature and annealing time were investigated in the ranges of 850–1000 °C and 15–90 min, respectively, using a laboratory furnace. After calcination, the clinker was crushed and leached in deionized water at 90 °C for 30 min. The performance of the process was evaluated in terms of lithium recovery, defined as *R*(Li) = *n*_Li,aq_/*n*_Li,tot_, where *n*_Li,aq_ represents the amount of lithium in the aqueous leachate after leaching of the clinker and *n*_Li,tot_ corresponds to the total amount of lithium introduced into the process. Exact formulations of the mixtures and their pretreatment, together with the detailed calcination and leaching procedures, are provided in the SI, Table S9.

The inherent variability in the overall experimental procedure was assessed under repeatability conditions using a reference mixture. The repeatability experiment gave a mean lithium transfer efficiency of 96.78%, with a 95% confidence interval of ±5.31 percentage points. Additional details concerning the repeatability test and the statistical evaluation of the experimental data are provided in the SI, Table S10.

### 2.3. Characterization and chemical analysis

Crystalline structure and phase evolution of the investigated materials were examined by X-ray diffraction (XRD) using a Bruker D8 Advance ECO diffractometer. The bulk elemental composition of the solid samples was determined by X-ray fluorescence spectroscopy (XRF) using a Bruker S8 TIGER spectrometer. Morphology and microstructure of the samples were studied using scanning electron microscopy (SEM) with a JEOL JSM-IT500HR instrument. A TGA Q500 analyzer was used for thermogravimetric measurements. Specific surface area and pore characteristics were determined by gas sorption using a Quantachrome AutosorbiQ instrument and analysed by the BET method. Elemental concentrations in solid samples and aqueous leachates were determined by inductively coupled plasma mass spectrometry (ICP-MS; Agilent 7900). Prior to analysis, solid samples were decomposed by microwave digestion using an Anton Paar Multiwave 5000 system in a mixture of HCl : HNO_3_ (3 : 1) and HF. All samples were analyzed in duplicate. Further analytical details, including QA/QC measures, are provided in the SI, Chapter S2.6.

## 3. Results and discussion

**Fig. 1:**
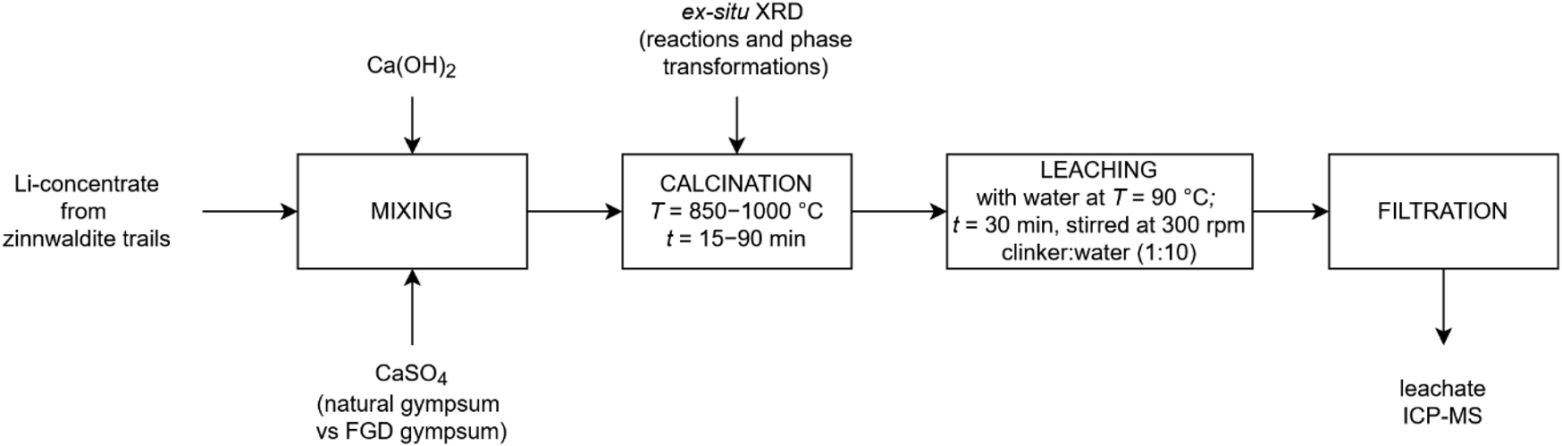
Schematic representation of the technological process for lithium recovery from the concentrate.

The process of lithium recovery was carried out in four steps: blending the Li concentrate with CaSO_4_ and Ca(OH)_2_, thermal treatment, aqueous leaching, and filtration. The process is schematically shown in Fig. 1. Among these steps, calcination is the key operation, as it governs the conversion of lithium from the original silicate matrix into water-soluble species that can subsequently be recovered by leaching. In simplified terms, the thermal treatment may be viewed as a sulphation – decomposition process, in which lithium is transferred into soluble lithium sulfate, whereas the silicate matrix is converted into poorly soluble calcium-containing phases. The dissolved lithium is then separated from the solid residue by water leaching and the resulting lithium-bearing solution is subjected to purification. The efficiency of the whole process depends on the quality of the Li-bearing feed as well as on the nature of the Ca- and sulfate-bearing phases.

### 3.1. Characterization of lithium concentrate

**Fig. 2:**
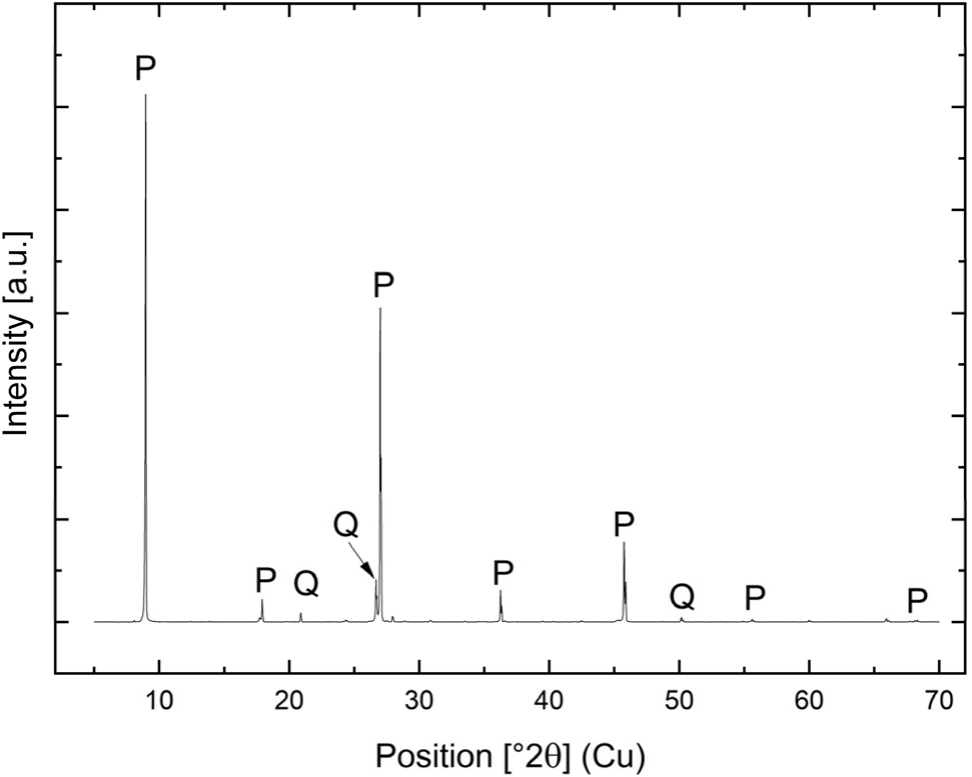
X-ray diffraction pattern of the lithium concentrate from the Cinovec tailings deposit with the identified crystalline phases, where P – polylithionite and Q – quartz. Individual structural database patterns are given in Fig. S1 in the SI.

The lithium-bearing feed used throughout this work was a zinnwaldite concentrate originating from the Cinovec tailings deposit. Table 2 shows the average composition of the lithium concentrate; only the main elements are included (full compositional analysis is presented in Table S1 in the SI). The lithium content in the concentrate was about 3500 mg kg^−1^ (0.35 wt%), and this was taken as the base value for the lithium recovery experiments. The analyses further showed that the material was dominated by the aluminosilicate matrix, with SiO_2_, Al_2_O_3_, K_2_O and Fe_2_O_3_ as the principal components, whereas CaO and MnO were present only at minor levels. It is worth noting the considerable rubidium content in the concentrate, corresponding to approximately half of the lithium content, which is consistent with previous observations for zinnwaldite-bearing material.^25^ Rubidium does not form separate, distinct crystalline phases that can be detected by XRD (Fig. 2). Instead, it is firmly bound within the mica crystal structure *via* isomorphic substitution. Because rubidium has a larger ionic radius, it substitutes for potassium in the interlayer positions of the silicate structure, creating strong chemical bonding.

**Table 2:** Multielement ICP-MS analysis of the lithium concentrate

Element	Concentration^a^ (mg kg^−1^)	Element	Concentration (mg kg^−1^)
Al	14 100 (335)	Mn	4700 (161)
As	45.6 (3.49)	Pb	170 (8.78)
B	2280 (343)	Rb	1630 (24.8)
Be	11.9 (4.93)	Sb	0.55 (0.05)
Cr	4.38 (0.20)	Se	2.39
Fe	43 800 (1120)	Ti	148 (9.17)
K	4270 (513)	Tl	28.6 (1.20)
Li	3520 (435)	Zn	373 (1.73)

a The reported concentrations are arithmetic means of independent analyses of duplicate samples taken from the homogenized concentrate. Values are rounded to 3 significant figures, standard errors of the mean are given in the parentheses. Extended version of the table can be found in the SI as Table S1.

According to XRD analysis (Fig. 2), the concentrate consisted predominantly of Li mica from the zinnwaldite/polylithionite series. Two phases were identified: dominant polylithionite (ferroan) and minor quartz. This structure is similar to that reported for other zinnwaldite concentrates from the Cinovec deposit.^5,6^

### 3.2. Characterization of natural and FGD gypsum

**Fig. 3:**
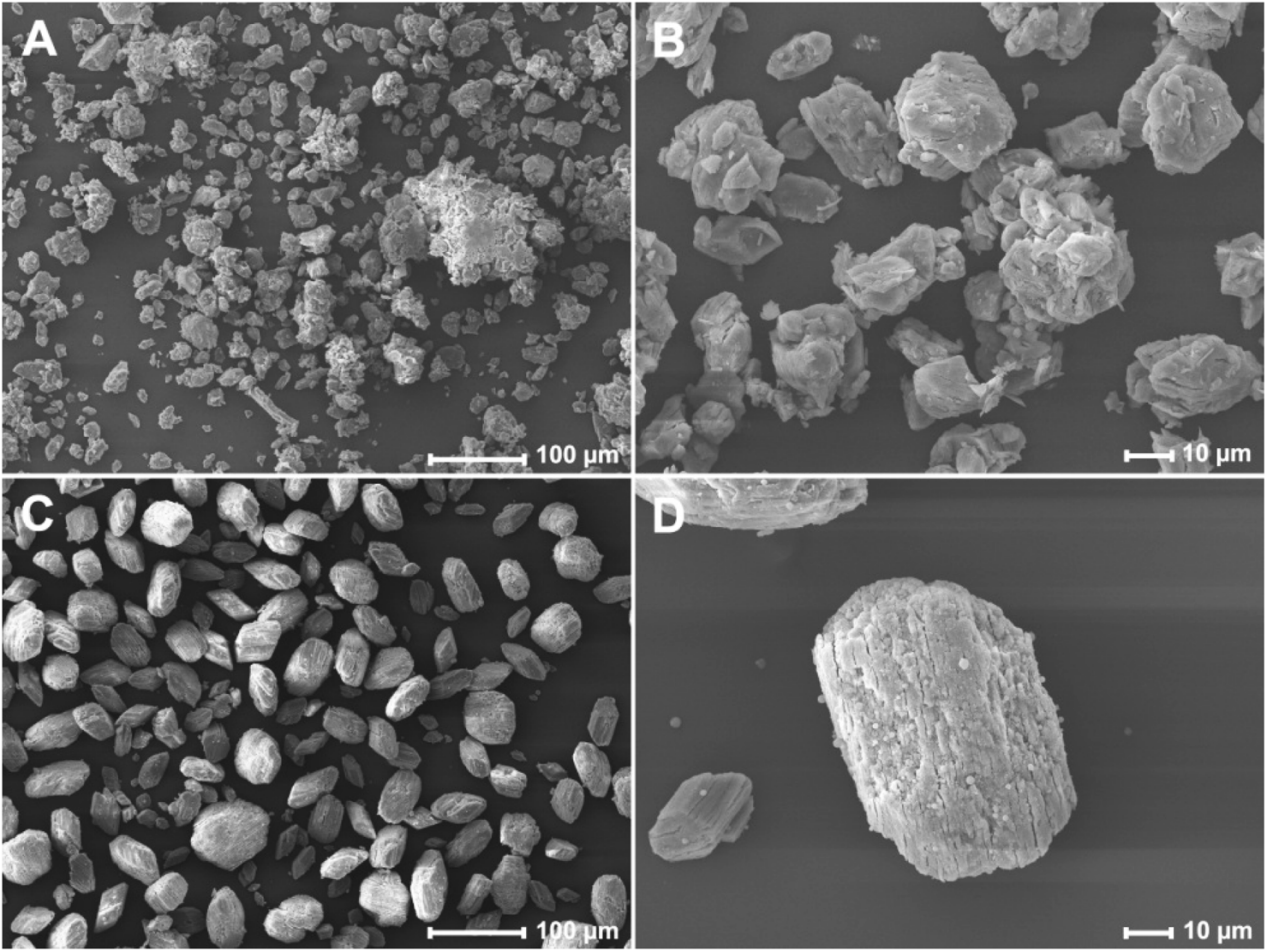
SEM micrographs of (A and B) natural gypsum after ball milling, and (C and D) FGD gypsum.

The main differences between natural gypsum and FGD gypsum were found in their morphology, homogeneity/heterogeneity, Ca content, and the content of some impurities (Si, Al and Fe; Table 3). The FGD gypsum was slightly richer in Ca and S (Table S6) than the natural gypsum (Table S4). The higher purity of FGD gypsum is also consistent with the order-of-magnitude lower contents of Al, Si and Fe (Chapters S1.2 and S1.3 in the SI). As can be seen from the SEM micrographs (Fig. 3), natural gypsum, after ball milling, consisted of sharp-edged, irregular fragments with a broader particle-size distribution of ∼39–60 µm (Table 3), whereas the FGD gypsum contained platy-to-acicular crystals with a narrower size distribution of ∼45–48 µm, consistent with precipitation-driven growth. Although the median particle diameters do not differ dramatically (Table 2), the presence of fine particles in natural gypsum increased its surface area, which may affect its reactivity.^23,26,27^

**Table 3:** Properties of natural and FGD gypsums. Detailed compositional and granulometric data are provided in the SI, Chapters S1.2 and S1.3

Property	Natural gypsum	FGD gypsum
LOI, 900 °C (%)^a^	9.69	6.91
Surface area (m^2^ g^−1^)	17.2	8.3
Particle size (µm)	39.02 D(0.5); 59.94 D(4.3)	44.64 D(0.5); 47.51 D(4.3)
Ca (wt%)	22.6	27.2
S (wt%)	16.8	22.4
Main impurities (wt%)	Si (4.33); Al (1.60); Fe (0.94)	Si (0.34); Al (0.19); Fe (0.10)

a loss on ignition (TG); *D*(0.5) = median particle diameter; *D*(4.3) = volume-weighted mean diameter.

**Fig. 4:**
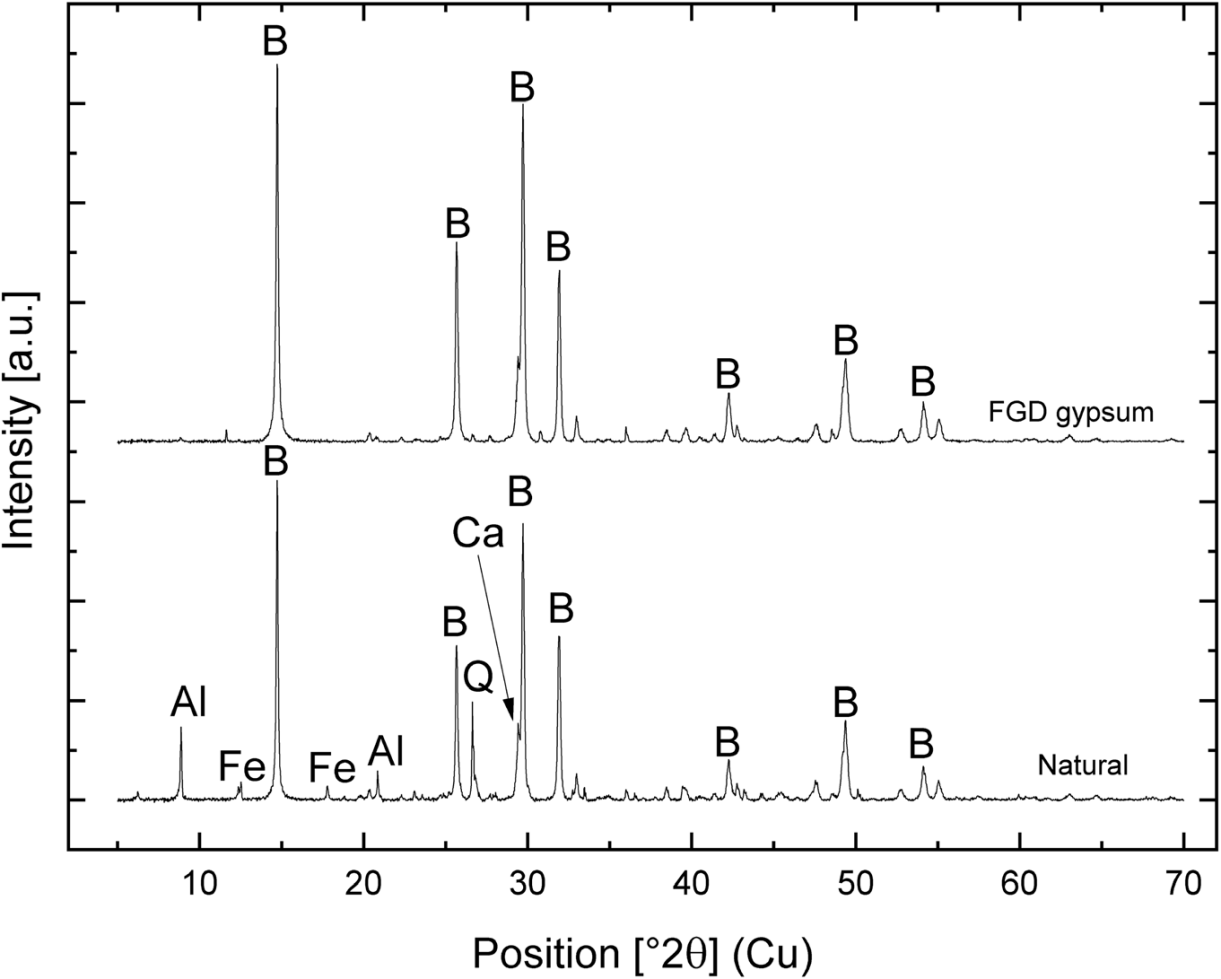
X-ray diffractograms of natural gypsum and FGD gypsum. Peak assignment: B – CaSO_4_·½H_2_O (bassanite), Q – SiO_2_ (quartz), Ca–CaCO_3_ (calcite), Al–Al_2_Si_2_O_5_(OH)_4_·5H_2_O (halloysite), Fe–Fe(SO_4_)(OH)(H_2_O)_2_ (butlerite). Individual structural database patterns are given in Fig. S4 in the SI.

X-ray diffraction (Fig. 4) showed that the natural gypsum was composed predominantly of calcium sulfate hemihydrate (CaSO_4_·½H_2_O; bassanite), together with detectable silicate impurities, particularly quartz (SiO_2_), minor aluminosilicate phases, and carbonate impurity (CaCO_3_). The FGD sample was dominated by bassanite. The lower impurity content (Table 3) and the dominant presence of a single crystalline phase in FGD gypsum may favour more reproducible dehydration and reaction pathways during heating.

### 3.3. Effect of calcination conditions on lithium recovery

**Fig. 5:**
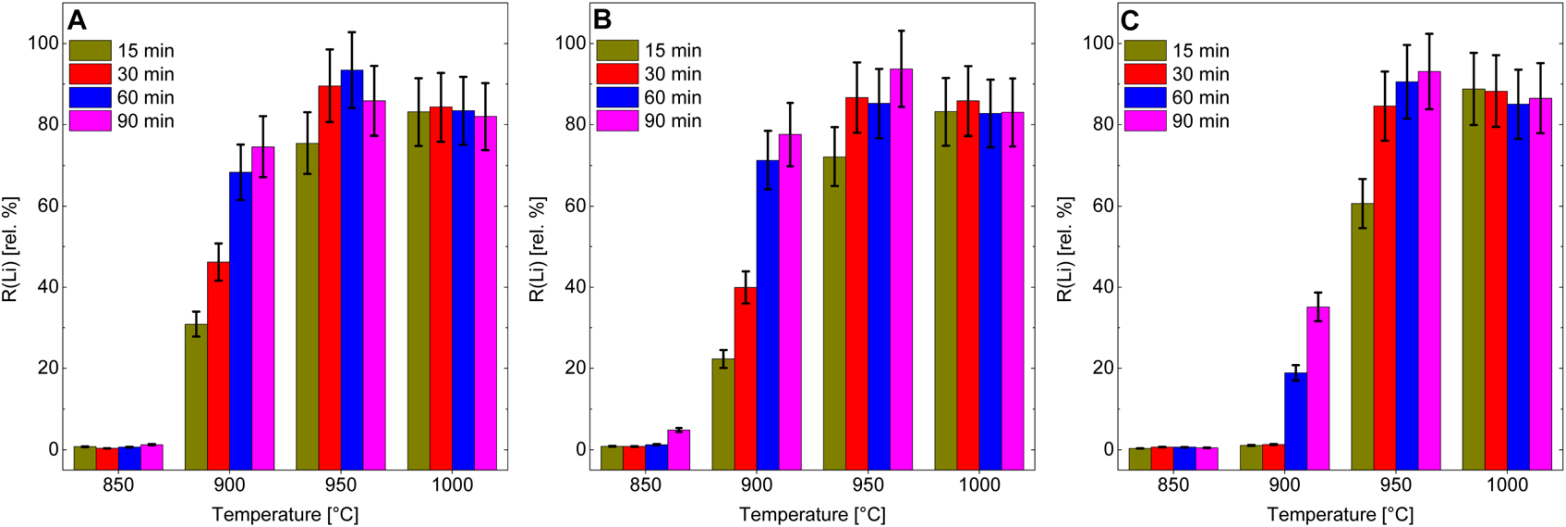
Dependence of lithium recovery on temperature and time of calcination for various reaction mixtures. (A) Reference reaction mixture of laboratory chemicals; (B) mixture containing natural gypsum; and (C) mixture containing FGD gypsum. Error bars were estimated from a repeatability experiment and correspond to the overall uncertainty of *ca.* 10% relative (coverage factor *k* = 2).

The effects of calcination temperature and annealing time on lithium recovery were investigated over the temperature range 850–1000 °C and the annealing time range 15–90 min, respectively (Fig. 5). After calcination, the product was ground and subsequently leached in water at 90 °C. The experimental arrangement ensured that the observed differences between the experimental series were primarily attributable to the origin and physicochemical properties of the gypsum materials.

The dependencies in Fig. 5 reflect a complex interplay of solid-state reactions during calcination with subsequent formation of lithium sulfates as water-extractable phases. The temperature interval between 900 and 950 °C corresponds to the main transition from poorly extractable to readily leachable Li forms with an extraction efficiency exceeding 80%.

Overall, the data point to a relatively narrow operational window in which the conversion of Li into water-leachable phases is maximised, whereas more intensive thermal treatment does not provide a further benefit and may even promote secondary incorporation of Li into less accessible solid phases.

For the reference mixture (Fig. 5A), the system composed of Li concentrate, laboratory-grade CaSO_4_ and Ca(OH)_2_ served as a benchmark against which the industrial gypsum sources could be evaluated. Lithium recovery increased with calcination temperature and annealing time, peaking at about 95.5% in a relatively narrow region near 950 °C and 30–90 min. A further increase in temperature to 1000 °C or prolonging the treatment did not provide a clear practical benefit, and in some cases recovery slightly decreased.

For the mixture containing natural gypsum (Fig. 5B), similar dependencies of Li recovery on temperature and annealing time were observed. The most efficient annealing temperature was 950 °C for an annealing time of 90 min, resulting in lithium recovery of 93.7%. This slight increase in annealing time may be related to the higher impurity content and broader particle-size distribution of the natural gypsum (see Fig. 4 and Table 3).

For FGD gypsum, Li recovery remained technologically negligible at 850 °C for 15 and 30 min, then increased to 20 and 35% for 60 and 90 min, respectively. At 950 °C, the recovery was much more strongly annealing-time dependent than in the previous cases and reached 93.1%, *i.e.* a value comparable to that for the natural gypsum. Increasing the temperature to 1000 °C caused a decrease in Li recovery. Somewhat different behaviour was observed ed for the FGD gypsum mixture at 900 °C, where the lithium recovery is distinctly lower than that obtained with laboratory-grade calcium sulphate and natural gypsum. This difference suggests that the lower-temperature boundary of the processing window is more sensitive to the phase composition and reactivity of the calcium sulphate source. In the case of FGD gypsum, the formation of the reactive calcium-containing intermediate phases appears to be delayed, resulting in lower apparent reaction rates and slower lithium release. However, this difference becomes much less pronounced at higher temperatures, indicating that FGD gypsum follows the same overall activation pathway but requires slightly more severe thermal conditions to achieve comparable conversion. This observation supports the practical conclusion that FGD gypsum is a viable calcium sulphate source, while also defining the limits of its use near the lower-temperature edge of the optimal processing window.

It is worth noting that, accompanying lithium, the dissolution of rubidium into solution remained low in all cases, approximately 8–10%. This suggests that rubidium behaved differently from lithium during the calcination – leaching cycle and could be recovered by a different activation route, for example chlorination roasting.^25^ The transfer of Ca into solution was low in all cases (approx. 2.2–2.5%), consistent with the predominantly insoluble nature of the calcium phases in the clinker. In contrast, potassium exhibited a higher degree of transfer into solution (approx. 29–33%).

Calcination proceeds as a series of parallel reactions in a heterogeneous multicomponent system, for which an exact stoichiometric description is difficult because of the formation of multiple crystalline and possibly glassy phases. For a general description, the following reaction schemes are typically considered during calcination at 950 °C: (i) decomposition of calcium hydroxide, Ca(OH)_2_ → CaO + H_2_O↑; (ii) binding of Si/Al into calcium phases, which can schematically be written as Al–Si structure of mica + CaO → Ca-silicates + Ca-aluminates; and (iii) conversion of lithium into the sulfate (water-soluble) phase, *i.e.* Li bound in mica + SO_4_^2−^ → Li_2_SO_4_ (or LiKSO_4_). The calcination step results in the formation of a sulfate phase, in which lithium is released from the original mica lattice; in the concentrate, the characteristic phase is primarily Li_2_SO_4_ or LiKSO_4_, depending on the local chemical composition.

**Fig. 6:**
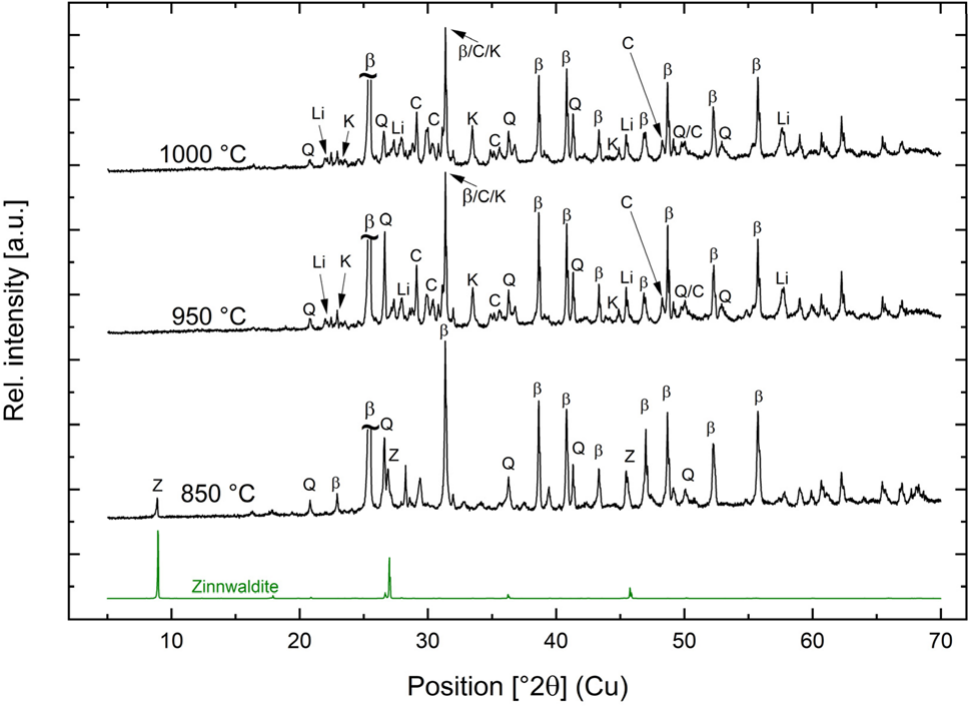
X-ray diffractograms of calcined samples with FGD gypsum at variable temperatures and 90 min annealing. Labelling: Z – zinnwaldite, β-anhydrous β-CaSO_4_, Q – SiO_2_, Li–Li_2_SO_4_, K – LiKSO_4_, and C – cuspidine. The intensity of the most pronounced β-CaSO_4_ peak at 25° is limited to improve figure clarity during scaling. Individual structural database patterns are given in Fig. S13, S14 and S15 in the SI.

The XRD analysis of the calcined products provided evidence of phase transformations occurring during thermal treatment. The diffractograms in Fig. 6 belong to the FGD gypsum mixture; however, similar trends were observed for all three mixtures. In general, a four-stage reaction sequence occurs:

1. Below 900 °C, zinnwaldite was thermally unstable and became essentially decomposed around 900 °C, which is consistent with the literature.^13^ CaSO_4_ did not react, which is also consistent with the negligible leaching efficiency observed in Fig. 5, as Li-bearing sulfate phases had not yet formed. The original bassanite (CaSO_4_·½H_2_O) transformed to anhydrous β-CaSO_4_.^28^

2. For intermediate annealing temperatures of 900–950 °C, solid-state reactions may be initiated at mica–sulfate grain interfaces. K^+^ and Li^+^ ions are progressively released from their positions in the crystalline lattice, combine with sulfate ions, and form LiKSO_4_ and Li_2_SO_4_. Fluorine is likely retained in Ca-bearing solid phases; under these conditions, cuspidine was identified by XRD among the calcination products (Fig. 6).

3. At 950 °C, nearly complete mica conversion was achieved. All accessible lithium was present in the form of sulfates (Fig. 6), and the lithium recovery reached its maximum of about 93% (Fig. 5).

4. At 1000 °C, the increased temperature promoted the creation of a more compact aluminosilicate refractory phase, which may incorporate some lithium. Lithium-containing grains may be covered by glassy phases, which reduces the availability of lithium. Impurities in the feed (Table 3) may enhance this effect. Sintering of the calcine further reduces leachability.

#### 3.3.1. Comparative assessment

A comparison of Fig. 5A–C shows that the three investigated mixtures can achieve similar lithium recovery, but within different effective calcination windows defined by temperature and time. The reference mixture reached near-optimal performance at about 950 °C and 60 min, whereas both mixtures prepared with industrial gypsum materials generally required an annealing time of about 90 min to achieve comparable results.

These differences should, however, be interpreted with caution. Under repeatability conditions, the relative standard deviation of *R*(Li) for the reference mixture was 5.31% (relative), and the expanded relative uncertainty of *R*(Li) may therefore be estimated at approximately 10% (*k* = 2). Minor differences between neighbouring data points should not therefore be overinterpreted. A further source of uncertainty is associated with the heating ramp preceding the isothermal hold, which is difficult to avoid in laboratory experiments and is likewise inherent to most industrial thermal treatment units (*e.g.* rotary kilns). For this reason, the transfer of the present results to industrial practice should be verified during process scale-up.

The present results should be interpreted not only in terms of the maximum lithium recovery achieved under a given set of conditions, but also in terms of process robustness with respect to the sulfate-bearing additive. From this perspective, the key finding is that gypsum-based materials, including FGD gypsum, remain applicable without a fundamental deterioration of performance. This is important because it suggests that the process is not restricted to model laboratory reagents but may be applicable to more practically relevant raw materials. In particular, the use of FGD gypsum introduces a circularity-related aspect, as it enables the valorization of an existing industrial by-product stream within the same general process concept.

#### 3.3.2. Kinetic interpretation of the calcination window

The temperature time dependencies in Fig. 5 indicate that lithium recovery is governed by competing processes occurring during calcination. In the first approximation, the formation of water-leachable lithium sulfate phases can be described by a pseudo-first-order model:

1 *R*(*t*) = *R*_∞_(1 −exp(−*k*_f_*t*))

where *R*_∞_ is the maximum attainable recovery and *k*_f_ is the apparent rate constant of lithium release and sulfate formation. The increase in recovery with temperature and time up to ∼950 °C is consistent with thermally activated solid-state reactions at mica–sulfate interfaces.

The existence of a maximum in *R*(Li) suggests that a secondary process becomes significant at longer times or higher temperatures. This behaviour can be described by a simple formation–deactivation model (Bateman function):

2 *R*(*t*) = *R*_∞_(1 −exp(−*k*_f_*t*)) exp(−*k*_d_*t*)

where *k*_d_ represents an apparent rate constant associated with lithium reincorporation into refractory phases, matrix densification, or formation of diffusion barriers.

Within this framework, the observed narrow processing window reflects a balance between lithium mobilization (*k*_f_) and its subsequent loss of accessibility (*k*_d_). The potential increase of *k*_d_ at higher temperatures, associated with higher aluminosilicate and possible glass-phase contents, thereby increasing diffusion barriers (Fig. 6), provides a tentative explanation for the slight decrease in recovery observed at 1000 °C. This simplified model is not intended for rigorous fitting but provides a physically consistent interpretation of the experimentally observed trends and supports the identification of optimal calcination conditions. It is also useful in the identification of differences in the calcination/extraction behaviour – some examples are given in the SI. The supplementary kinetic analysis using the Bateman-type model supports the existence of a finite processing window. The model describes the formation-dominated increase in lithium recovery for most mixtures, while the slight decrease observed for FGD gypsum at 1000 °C is consistent with a slow loss of leachability during prolonged calcination. In contrast, the FGD gypsum mixture at 900 °C shows a sigmoidal response, indicating an induction period and suggesting that this temperature is close to the lower limit required for efficient formation of leachable lithium phases.

### 3.4. Effect of the pre- and post-calcination treatment on lithium recovery

Particle size is commonly reported as an important parameter affecting lithium extraction from mineral materials; in many systems, finer particles promote higher extraction because of their larger specific surface area, shorter intraparticle diffusion paths, and improved contact between the phases participating in the reaction.^29^ This was also confirmed in a recent study.^11^

For the system with zinnwaldite concentrate as the Li-bearing substrate, relevant literature data are scarce. Additional experiments were therefore carried out to evaluate the effects of (i) milling of the zinnwaldite concentrate prior to calcination and (ii) milling of the calcined clinker prior to leaching on Li recovery. The aim was to distinguish between the effect of feed particle size on the extent of solid-state conversion during calcination and the effect of clinker comminution on subsequent leaching, solid–liquid separation, and apparent Li recovery. The zinnwaldite concentrate used in the standard mixture was subjected to stepwise comminution to obtain three particle-size levels. The unmilled concentrate had *D*(0.5) ≈ 218 µm and *D*(4.3) ≈ 258 µm (Table S13). After the first milling step, these values decreased to 80.9 and 132 µm, respectively, and after the second step to 22.4 and 44 µm, respectively. Each material was then processed under identical calcination and leaching conditions, and Li recovery, *R*(Li), was determined from the Li concentration in the leachate. The particle-size distribution was characterised by *D*(0.5) and *D*(4.3), corresponding to the median diameter and the volume-weighted mean diameter, respectively. For more detailed particle-size distributions, see Fig. S11 in the SI.

Milling of the concentrate had a strong positive effect on Li recovery. *R*(Li) increased from ∼81.7% for the unmilled material to ∼91.1% after the first milling step and reached ∼98.6% after the second step (Table S14). This trend can be attributed to improved homogenization of the feed with the Ca-bearing additives, an increased reactive interfacial area, and shorter solid-state diffusion distances during calcination, all of which favour more complete decomposition of zinnwaldite and more efficient conversion of Li into water-soluble sulfate phases.

In a complementary set of experiments, the calcined clinker was milled prior to leaching to examine whether further size reduction would enhance Li release. A coarser clinker fraction with *D*(0.5) ≈ 80 µm was compared with an ultrafine material produced by intensive milling, with *D*(0.5) ≈ 4.7 µm (Table S17). The leaching and analytical procedures were kept unchanged, and Li recovery was again evaluated from the filtrate composition.

In contrast to the beneficial effect of concentrate milling, excessive milling of the clinker decreased the apparent Li recovery from ∼97.8% for the coarser clinker to ∼87.2% for the ultrafine material (Table S19). This decrease is unlikely to reflect a lower intrinsic Li leachability; rather, it is most plausibly a filtration artefact associated with solid–liquid separation. Ultrafine particles form a dense filter cake during vacuum filtration, which hinders liquor percolation and increases retention of Li-bearing solution within the cake. As a result, the Li concentration measured in the filtrate may underestimate the actual amount of Li dissolved during leaching. These results indicate that fine milling of the concentrate prior to calcination is beneficial, whereas excessive comminution of the clinker prior to filtration should be avoided. Mild and standardised clinker disintegration is therefore recommended to ensure reproducible leaching and reliable recovery data.

## 4. Conclusions

This work demonstrated that natural gypsum and FGD gypsum can serve as practically relevant sulfate-bearing additives in gypsum-assisted lithium recovery from a zinnwaldite-bearing concentrate derived from the Cinovec tailings repository. Under optimal conditions, lithium recovery reached about 93% for all examined additives. The principal operating window was centred around 950 °C with an annealing time of 60–90 min. It is governed by zinnwaldite decomposition, complete conversion of lithium into extractable sulfate phases, and the formation of an insoluble residual matrix consisting of cuspidine, aluminosilicates and possibly glassy material. Longer annealing times did not significantly improve lithium recovery; in some cases, they even led to a slight decrease, likely because of densification of the residual refractory matrix under more severe temperature time conditions, which may partially immobilise lithium. Milling of the concentrate prior to calcination had a strong positive effect on Li recovery, whereas excessive comminution of the clinker prior to filtration deteriorated the apparent lithium recovery. A relatively narrow operating window, comparable to that reported in earlier studies employing laboratory-grade reagents as calcination additives, indicates that insights from previous laboratory research can provide a solid foundation for scaling up the gypsum-based process. At the same time, the results suggest that the operating window is broad enough to allow the use of technically relevant gypsum-based additives, including FGD gypsum. The work extends beyond practical process engineering by providing fundamental chemical insights into the phase evolution and kinetic constraints of utilizing heterogeneous secondary sulfates. The study highlights that the mobilization of critical materials, such as lithium, requires a complementary approach at the interface of chemistry, structural characterization and mineralogy. Therefore, these findings bridge laboratory studies of sulfate roasting with a more application-oriented flowsheet, in which an industrial by-product can replace reagent-grade CaSO_4_ without a substantial loss of lithium recovery. This enhances the practical relevance of the process and supports the valorization of an industrial by-product as a sulfate additive. The findings also provide a basis for further evaluation of other technically relevant sulfate-containing secondary materials.

## Conflicts of interest

There are no conflicts to declare.

## Supplementary Material

RA-016-D6RA04143D-s001

## Data Availability

Additional data are available from the corresponding author on reasonable request. The data supporting this article have been included as part of the supplementary information (SI). Supplementary information is available. See DOI: https://doi.org/10.1039/d6ra04143d.
