# Supplementary material for: Recovery of lithium from a zinnwaldite-bearing concentrate derived from tailings using commonly available flue-gas desulfurization gypsum
Source: RSC Adv. 2026 Jul 3;16(35):36241–51. doi: 10.1039/d6ra04143d (PMC13330580; doi:10.1039/d6ra04143d)
Supplement: RA-016-D6RA04143D-s001 [file RA-016-D6RA04143D-s001.pdf]

Supplementary Information (SI) for RSC Advances.
This journal is © The Royal Society of Chemistry 2026

Supplementary Information (SI)

Recovery of lithium from zinnwaldite tailings using commonly available flue-gas desulfurization gypsum

This Supplementary Information (SI) provides additional experimental details, characterization data, and analytical methods QA/QC supporting the main manuscript.

Content

Chapter S1. Materials and chemicals 5

Chapter S1.1. Lithium concentrate 5

Chapter S1.2. Natural gypsum from the Kobeřice mine near Opava 8

Chapter S1.3. FGD gypsum – Ledvice power plant 10

Chapter S1.4. Hydrated lime CL 90-S – Vápenka Čertovy schody, Tmaň 12

Chapter S2. Calcination and leaching experiments 13

Chapter S2.1. Adjustment of batch-mixture ratios 13

Chapter S2.2. Methodology of the metallurgical experiments 14

Chapter S2.3. Effect of concentrate particle size on lithium transfer efficiency 17

Chapter S2.4. Effect of clinker particle size on lithium transfer to solution 19

Chapter S2.5. Effect of calcination conditions on lithium transfer 20

Chapter S2.6. Elemental analysis of lithium concentrate and other calcination additives and products by ICP-MS 21

Chapter S3. Results of calcination experiments with various reaction mixtures 22

Chapter S4. Verification of reaction transformations by XRD analysis 23

Chapter S5. Appendix. Bateman-type model for lithium recovery from zinnwaldite 29

List of figures

Figure S1. X-ray diffraction pattern (XRD) of the lithium concentrate from the Cínovec tailings deposit with the identified crystalline phases indicated (PDF database). 6

Figure S2. SEM images lithium concentrate at different magnifications: (a) 200×, (b) 500×, (c) 1000×, (d) 10 000×. 7

Figure S3. Particle-size distribution of the lithium concentrate. 7

Figure S4. X-ray diffraction pattern (XRD) of Kobeřice natural gypsum after milling, supplemented with reference diffraction patterns of the identified phases (PDF database). 9

Figure S5. SEM images of Kobeřice natural gypsum after milling at different magnifications: (a) 200×, (b) 500×, (c) 1000×, (d) 10 000×. 9

Figure S6. Particle-size distribution of Kobeřice natural gypsum after mechanical size reduction by milling (mean of three measurements); the table lists the characteristic granulometric parameters. 10

Figure S7. X-ray diffractogram of FGD gypsum from the Ledvice power plant (sample 8276/2021) with reference diffraction lines of the identified phase indicated (bassanite – $CaSO_4 \cdot 0.5H_2O$). 11

Figure S8. SEM images FGD gypsum from the Ledvice power plant at different magnifications: (a) 200×, (b) 500×, (c) 1000×, (d) 10 000×. 11

Figure S9. Particle-size distribution of FGD gypsum (Ledvice power plant); the table lists characteristic values of the distribution parameters 12

Figure S10. Particle-size distribution and characteristic granulometric parameters of hydrated lime. 12

Figure S11. Particle-size distribution of the lithium concentrate for three particle-size variants. 17

Figure S12. Particle-size distribution of calcined clinker for variants without subsequent milling (1/D1) and after a single milling step in a planetary mill (1/D2). 19

Figure S13. XRD diffraction pattern of clinker from reference mixture (950 °C / 60 min), mixture 2 (950 °C / 90 min) and mixture 3 (950 °C / 90 min).the X-ray diffraction analysis was focused on

verifying the effect of calcination temperature on the course of reaction transformations in this mixture. 25
Figure S14. XRD diffraction pattern of clinker from FGD gypsum mixture at 850, 900. 950 and 1000 °C annealed for 90 min). 27
Figure S15. XRD diffraction pattern of clinker from FGD gypsum mixture at 950 °C for 15, 30, 60, and 90 min. 28
Figure S16. Dependencies of the lithium recovery on the time of calcination for selected mixtures and temperatures. 30

List of tables

Table S1. Elemental composition of the lithium concentrate by ICP-MS. 5
Table S2. Particle-size distribution of the lithium concentrate....8
Table S3. Specific surface area determined by the BET 8
Table S4. Elemental composition of natural gypsum from the Kobeřice mine by ICP-MS. 8
Table S5. Particle-size distribution of Kobeřice natural gypsum after mechanical size reduction by milling (mean of three measurements); the table lists the characteristic granulometric parameters. 10
Table S6. Elemental composition of FGD gypsum from the Ledvice power plant by ICP-MS. 10
Table S7. Particle-size distribution of FGD gypsum (Ledvice power plant); the table lists characteristic values of the distribution parameters. 12
Table S8. Particle-size distribution and characteristic granulometric parameters of hydrated lime. 13
Table S9. Adjustment of batch-mixture ratios according to the Ca content in the sulfate raw material used (wt. parts). 13
Table S10. Determination of the confidence interval of lithium transfer efficiency in parallel calcination–leaching experiments (mixture 1, n = 5). 15
Table S11. Mass balance of selected elements (Ca, K, Li, Rb) between the clinker, filtrate, and insoluble residue after leaching — parallel samples 1/V1 to 1/V5. 16
Table S12. Transfer of selected elements to solution at different degrees of mechanical treatment of the lithium concentrate (ZC) (15 g clinker charge; clinker-to-water ratio 1:10; temperature 90 °C; leaching time 30 min). 17
Table S13. Particle-size distribution of the lithium concentrate for three particle-size variants. 18
Table S14. Mass-balance evaluation of selected elements after clinker leaching – unmilled lithium concentrate. 18
Table S15. Mass-balance evaluation of elements after clinker leaching – 1× milled lithium concentrate. 18
Table S16. Mass-balance evaluation of elements after clinker leaching – 2× milled lithium concentrate. 18
Table S17. Particle-size distribution of calcined clinker for variants without subsequent milling (1/D1) and after a single milling step in a planetary mill (1/D2). 19
Table S18. Transfer of selected elements to solution as a function of mechanical treatment of the calcined clinker (6 g charge; clinker:water ratio = 1:10; temperature 90 °C; leaching time 30 min). 20
Table S19. Mass balance of selected elements (Ca, K, Li, Rb) between the clinker, filtrate, and insoluble residue after leaching — samples 1/D1 and 1/D2 20
Table S20. Mixing ratios and weighed amounts of the components used in the mixtures 21
Table S21. Lithium recovery for reference mixture as a function of calcination temperature and time. (The recovery calculated from the lithium balance and expressed in %) 22
Table S22. Lithium recovery for natural gypsum mixture as a function of calcination temperature and time. 23
Table S23. Lithium recovery for FGD gypsum mixture as a function of calcination temperature and time. 23
Table S24. Identified crystalline phases of clinkers from reference mixture, natural gypsum mixture and FGD gypsum mixture at maximum lithium transfer efficiency (XRD). 24
Table S25. Identified crystalline phases of clinkers from FGD gypsum mixture as a function of calcination temperature (calcination time 90 min, XRD). 26
Table S26. Model suitability and model parameters for selected systems 30

Chapter S1. Materials and chemicals

Chapter S1.1. Lithium concentrate

Table S1. Elemental composition of the lithium concentrate by ICP-MS.

Element	Sample-1 (mg/kg)	Sample-2 (mg/kg)	Mean (mg/kg)	SD	RSD (%)
Ag	<LOQ	<LOQ			
Al	14454,6	13785,4	14120,0	334,6	2,4
As	49,1	42,1	45,6	3,5	7,7
B	2618,9	1933,2	2276,05	342,9	15,1
Ba	<LOQ	<LOQ			
Be	16,8	7,0	11,9	4,9	41,2
Bi	<LOQ	<LOQ			
Ca	<LOQ	<LOQ			
Cd	<LOQ	<LOQ			
Co	<LOQ	<LOQ			
Cr	4,6	4,2	4,4	0,2	4,6
Cu	<LOQ	<LOQ			
Fe	44879,3	42643,4	43761,35	1117,9	2,6
K	4782,0	3755,3	4268,65	513,4	12,0
Li	3951,6	3082,7	3517,15	434,5	12,4
Mg	<LOQ	<LOQ			
Mn	4859,3	4536,9	4698,1	161,2	3,4
Mo	<LOQ	<LOQ			
Na	<LOQ	<LOQ			
Ni	<LOQ	<LOQ			
Pb	178,7	161,1	169,9	8,8	5,2
Rb	1652,4	1602,8	1627,6	24,8	1,5
Sb	0,6	0,5	0,55	0,05	9,1
Se	6,3	<LOQ			
Sr	<LOQ	<LOQ			
Ti	156,9	138,5	147,7	9,2	6,2
Tl	29,8	27,4	28,6	1,2	4,2
V	<LOQ	<LOQ			
Zn	374,7	371,2	372,95	1,7	0,5

NOTE: The table presents the results of the multielement ICP-MS analysis of the lithium concentrate. The reported concentrations correspond to the arithmetic mean of the ICP-MS determinations performed on the duplicate samples. The value "<LOQ" indicates a concentration below the limit of quantification of the method.

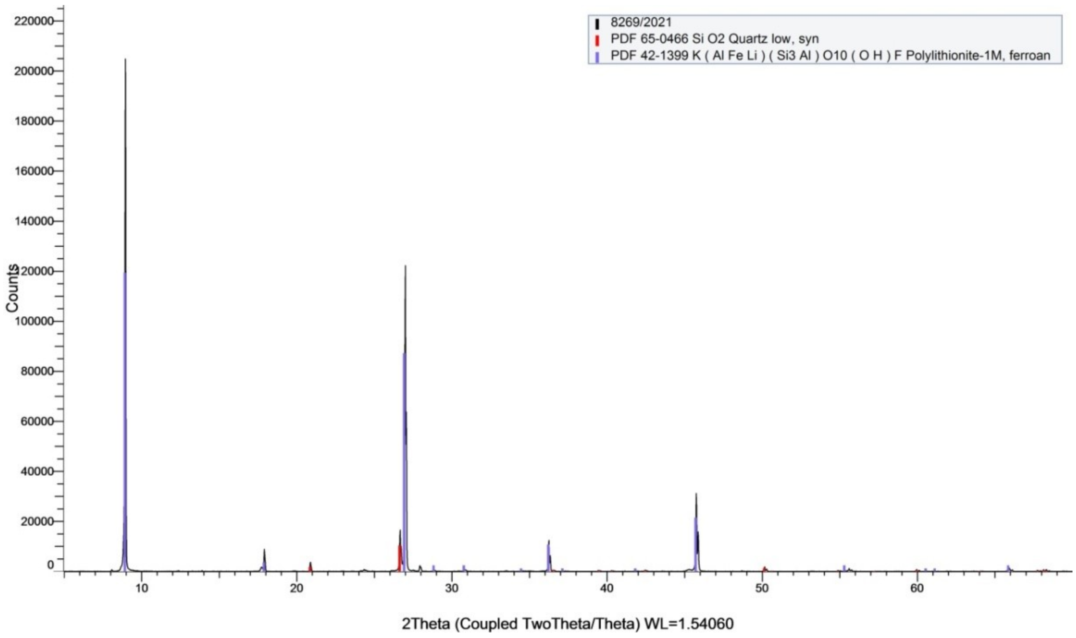

Figure S1. X-ray diffraction pattern (XRD) of the lithium concentrate from the Cínovec tailings deposit with the identified crystalline phases indicated (PDF database).

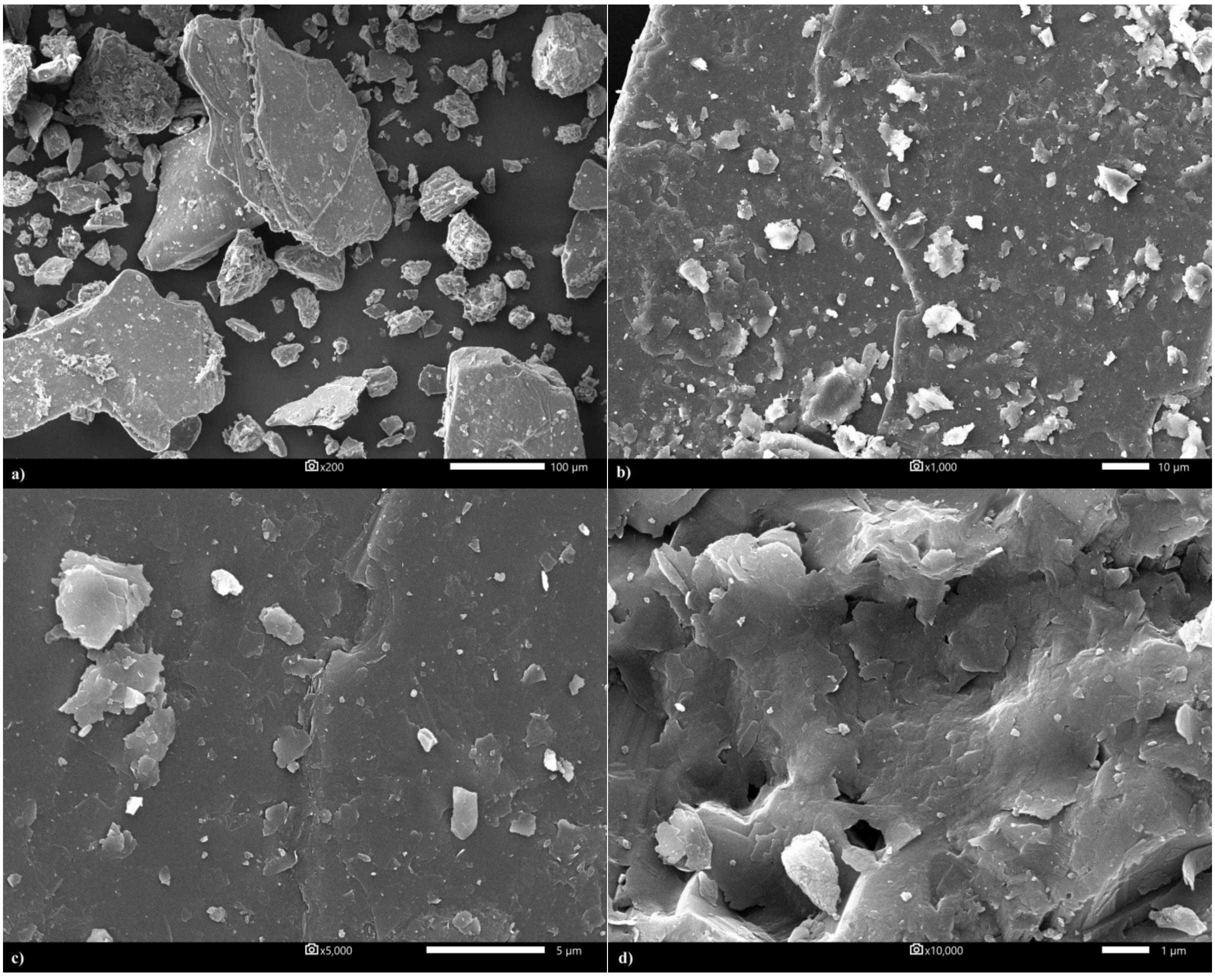

Figure S2. SEM images lithium concentrate at different magnifications: (a) 200×, (b) 500×, (c) 1000×, (d) 10 000×.

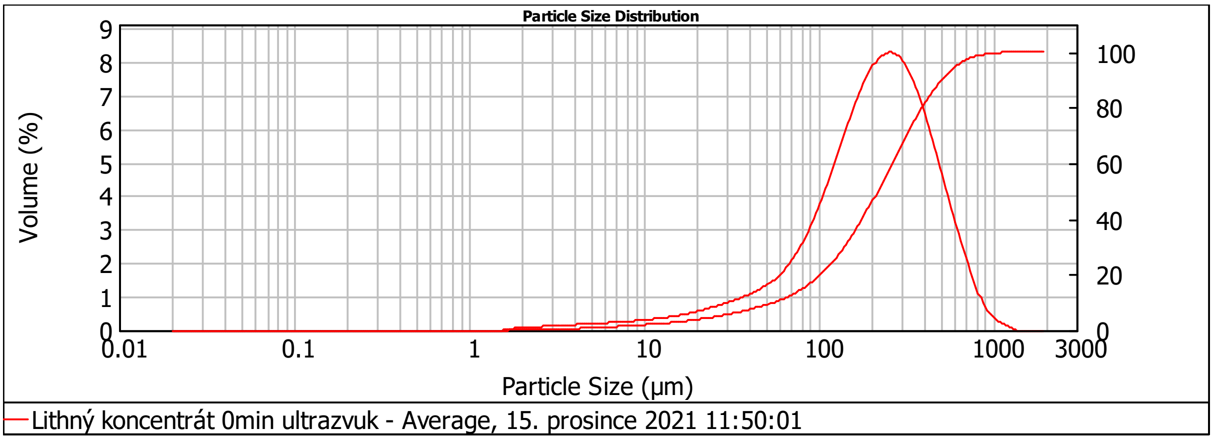

Figure S3. Particle-size distribution of the lithium concentrate.

Table S2. Particle-size distribution of the lithium concentrate.

D(0.1)	D(0.5)	D(0.9)	D(4.3)
55.090	218.401	510.133	257.631

Table S3. Specific surface area determined by the BET

Materials	Specific surface area (m²/g)
Lithium concentrate	4.10
Natural gypsum	17.20
FGD gypsum	8.30

Chapter S1.2. Natural gypsum from the Kobeřice mine near Opava

Table S4. Elemental composition of natural gypsum from the Kobeřice mine by ICP-MS.

Element	Concentration (mg/kg)	Element	Concentration (mg/kg)
Ag	< 5	Mg	1420.69
Al	2130.32	Mn	185.21
As	< 6	Mo	1.29
B	10.65	Na	76.18
Ba	39.71	Ni	8.08
Be	< 5	P	< 5
Bi	< 5	Pb	< 5
Ca	226168.18	S	167756.20
Cd	< 2	Sb	< 5
Co	2.58	Se	< 5
Cr	5.17	Sr	4227.80
Cu	8.40	Ti	47.80
Fe	7441.46	Tl	< 5
K	1069.00	V	< 2

NOTE: The value “<LOQ” indicates a concentration below the limit of quantification of the method.

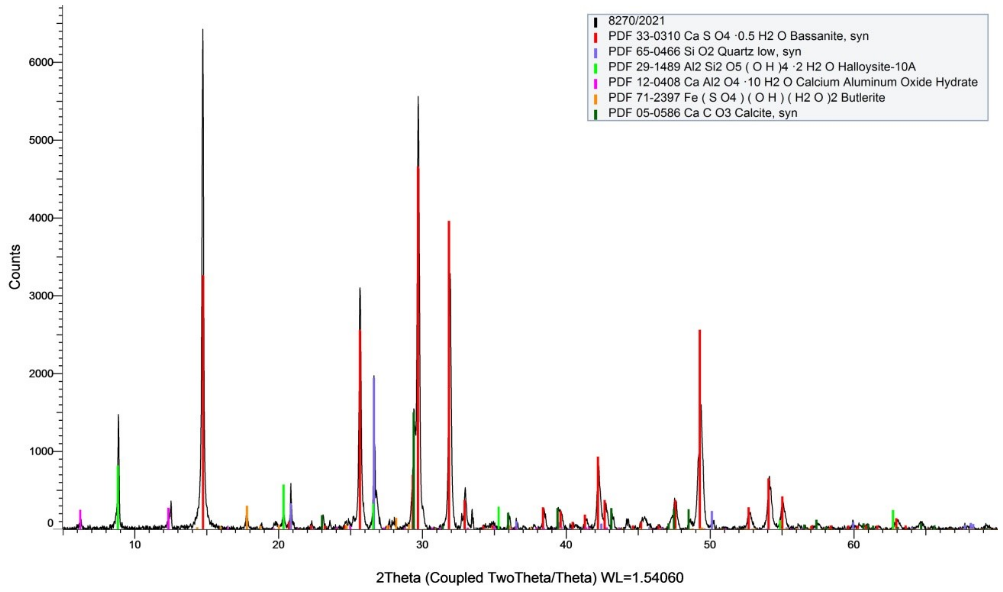

Figure S4. X-ray diffraction pattern (XRD) of Kobeřice natural gypsum after milling, supplemented with reference diffraction patterns of the identified phases (PDF database).

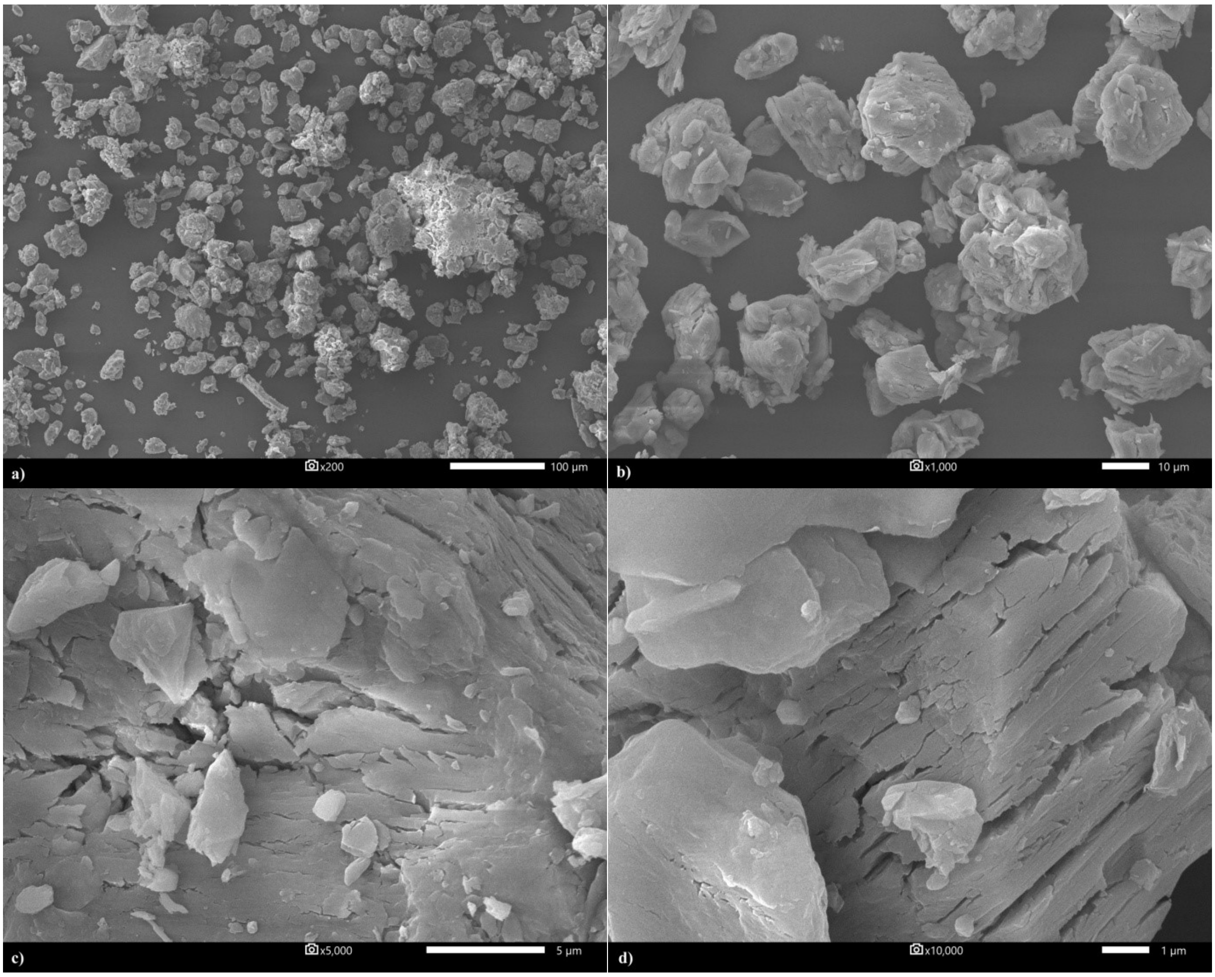

Figure S5. SEM images of Kobeřice natural gypsum after milling at different magnifications: (a) 200×, (b) 500×, (c) 1000×, (d) 10 000×.

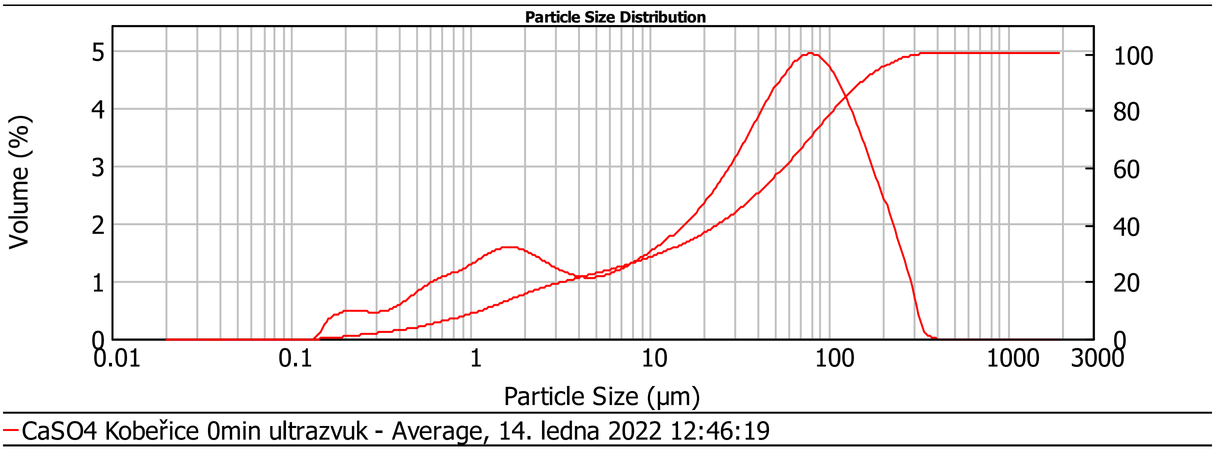

Figure S6. Particle-size distribution of Kobeřice natural gypsum after mechanical size reduction by milling (mean of three measurements); the table lists the characteristic granulometric parameters.

Table S5. Particle-size distribution of Kobeřice natural gypsum after mechanical size reduction by milling (mean of three measurements); the table lists the characteristic granulometric parameters.

Parametr	D(0.1)	D(0.5)	D(0.9)	D(4.3)
[μm]	1.16	39.02	153.93	59.94

Chapter S1.3. FGD gypsum – Ledvice power plant

Table S6. Elemental composition of FGD gypsum from the Ledvice power plant by ICP-MS.

Element	Concentration (mg/kg)	Element	Concentration (mg/kg)
Ag	< 5	Mg	238.30
Al	380.04	Mn	21.04
As	< 5	Mo	< 2
B	26.20	Na	151.74
Ba	5.91	Ni	< 2
Be	< 5	P	129.16
Bi	< 5	Pb	< 5
Ca	272393.70	S	223675.66
Cd	< 2	Sb	< 5
Co	< 2	Se	10.51
Cr	2.30	Sr	138.70
Cu	2.30	Ti	25.65
Fe	857.90	Tl	< 5
K	265.15	V	2.30

NOTE: The value "<LOQ" indicates a concentration below the limit of quantification of the method.

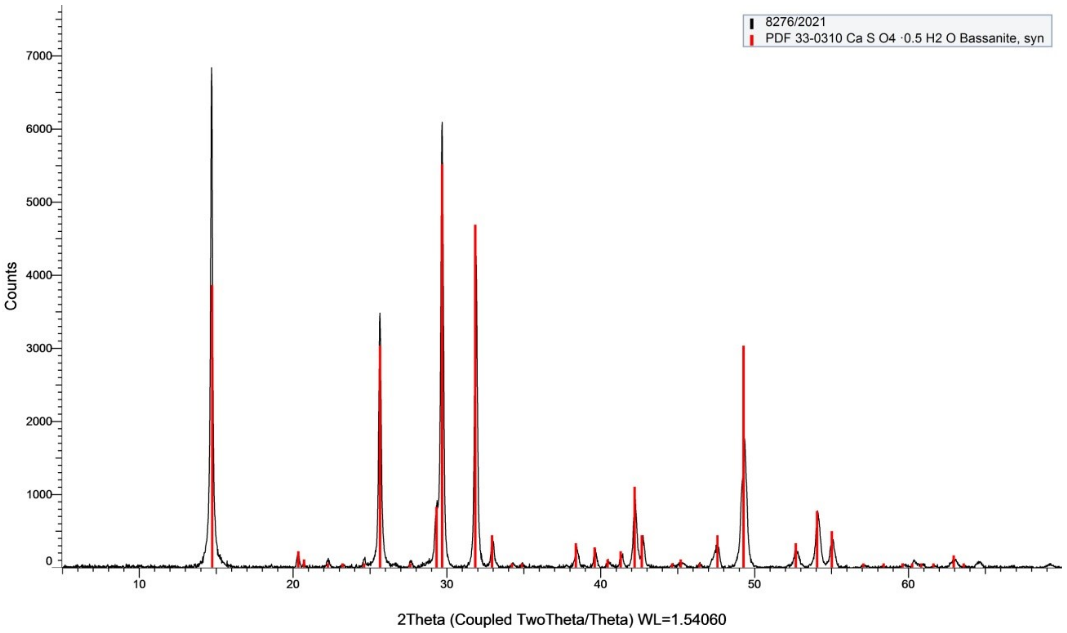

Figure S7. X-ray diffractogram of FGD gypsum from the Ledvice power plant (sample 8276/2021) with reference diffraction lines of the identified phase indicated (bassanite – $CaSO_4 \cdot 0.5H_2O$).

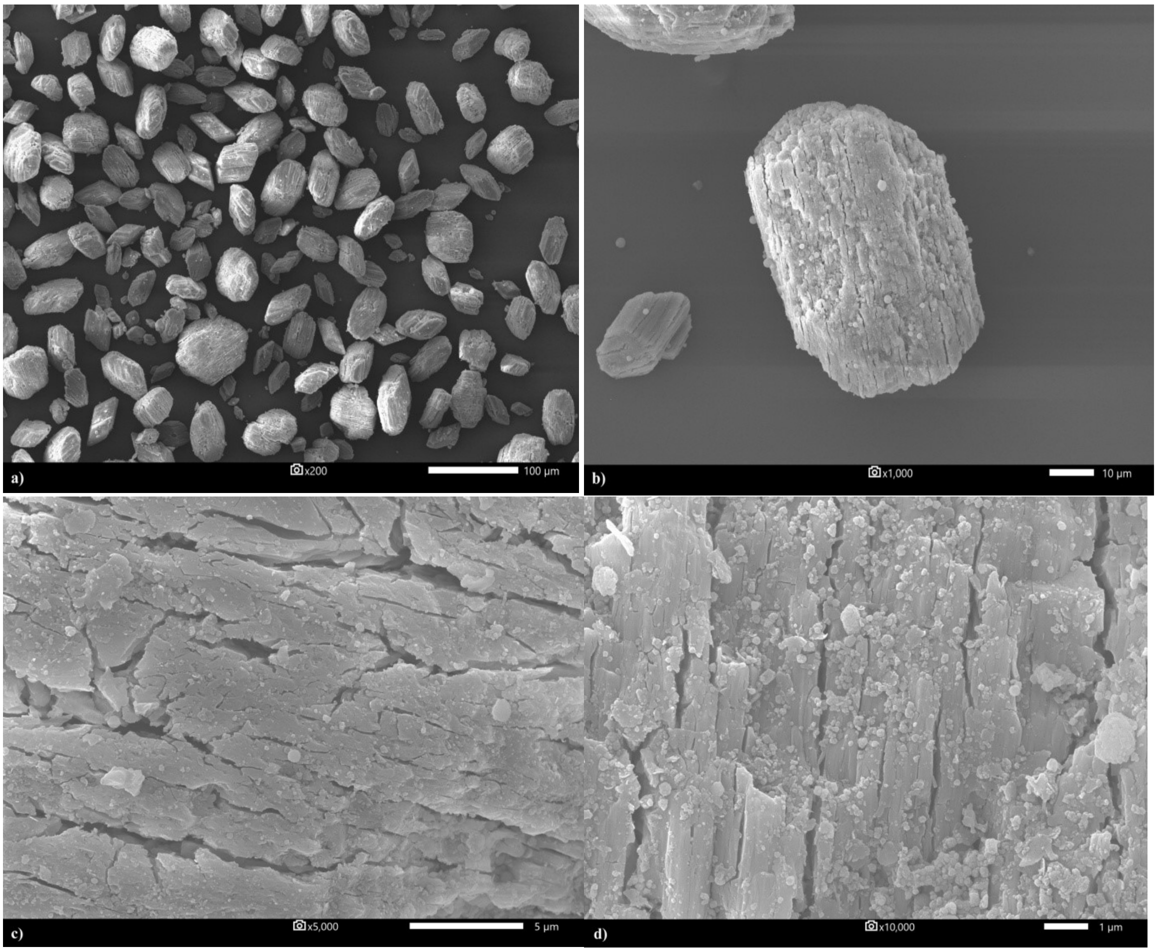

Figure S8. SEM images FGD gypsum from the Ledvice power plant at different magnifications: (a) 200×, (b) 500×, (c) 1000×, (d) 10 000×.

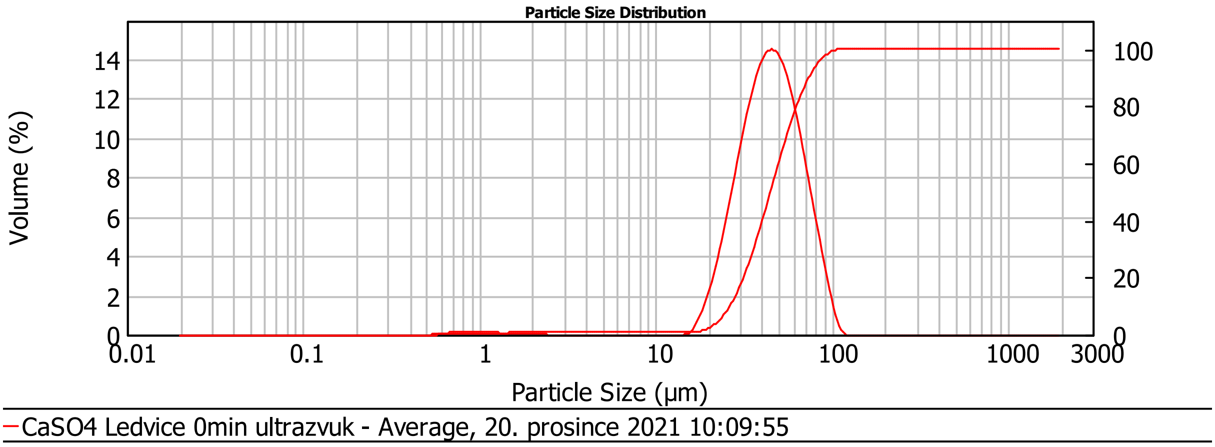

Figure S9. Particle-size distribution of FGD gypsum (Ledvice power plant); the table lists characteristic values of the distribution parameters.

Table S7. Particle-size distribution of FGD gypsum (Ledvice power plant); the table lists characteristic values of the distribution parameters.

Parametr	D(0.1)	D(0.,5)	D(0.9)	D(4.3)
[μm]	26.22	44.64	73.73	47.51

Chapter S1.4. Hydrated lime CL 90-S – Vápenka Čertovy schody, Tmaň

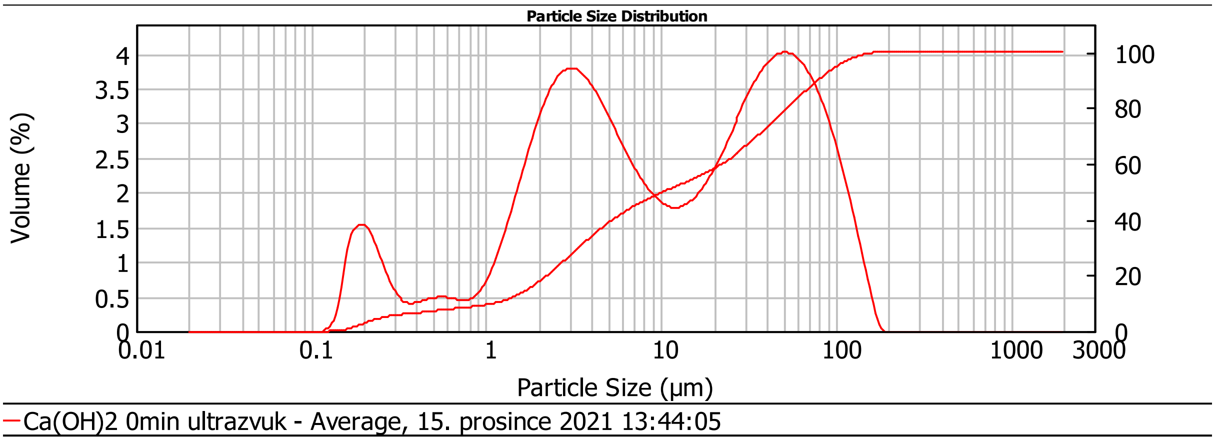

Figure S10. Particle-size distribution and characteristic granulometric parameters of hydrated lime.

Table S8. Particle-size distribution and characteristic granulometric parameters of hydrated lime.

Parametr	D(0.1)	D(0.5)	D(0.9)	D(4.3)
[μm]	1.11	10.23	79.30	27.70

Chapter S2. Calcination and leaching experiments

Chapter S2.1. Adjustment of batch-mixture ratios

Three variants of batch mixtures were used, differing in the type of sulfate raw material (laboratory-grade $CaSO_4 \cdot 2H_2O$, natural gypsum, and FGD gypsum). To maintain a comparable total amount of calcium in the batch, the mixing ratios were adjusted by changing the mass fraction of the sulfate component. The proportion of $Ca(OH)_2$ was kept constant in all mixtures because the CL 90-S hydrated lime used is a raw material with declared parameters according to the relevant standard classification. The lithium concentrate was also dosed at the same mass fraction in all mixtures.

Table S9. Adjustment of batch-mixture ratios according to the Ca content in the sulfate raw material used (wt. parts).

Mixture 1 (reference in the main text)	Ca content in the sulfate raw material (g/kg)	lithium concentrate (wt. parts)	Sulfate component: Synthetic ($CaSO_4 \cdot 2H_2O$) p.a. (wt. parts)	$Ca(OH)_2$ p.a. (wt. parts)
Reference laboratory mixture with $CaSO_4 \cdot 2H_2O$ gypsum	232.79	6	4.2	2
Mixture 2 (natural gypsum in the main text)	Ca content in the sulfate raw material (g/kg)	lithium concentrate (wt. parts)	Sulfate component: natural gypsum from the Kobeřice mine (wt. parts)	Hydrated lime CL 90-S (wt. parts)
Mixture with natural gypsum from the Kobeřice mine	226.16	6	4.3	2
Mixture 3 (FGD gypsum in the main text)	Ca content in the sulfate raw material (g/kg)	lithium concentrate (wt. parts)	Sulfate component: FGD gypsum from power plant Ledvice (wt. parts)	Hydrated lime CL 90-S (wt. parts)
Mixture with FGD gypsum from the Ledvice power plant	272.39	6	3.6	2

Note: The Ca content in the laboratory reference sulfate component was determined by calculation from the molar masses of $CaSO_4 \cdot 2H_2O$; the Ca content in Kobeřice natural gypsum and Ledvice FGD gypsum was based on ICP-MS determination. The proportions of $Ca(OH)_2$ and lithium concentrate were kept constant in all mixtures.

The batch mixtures were prepared by mixing of the individual components according to the specified ratios. The sulfate raw materials (natural gypsum and FGD gypsum) were pre-dried at 160 °C for 4 hours before use to remove adsorbed and weakly bound water. From the prepared and homogenized material, 20 g portions were weighed out and pressed at a force of 30 kN into tablets with a diameter of 40 mm. The prepared tablets were labeled with identification numbers, sorted by mixture type into separate closed containers, and stored under laboratory conditions until the calcination experiments were performed.

Chapter S2.2. Methodology of the metallurgical experiments

Calcination experiments were carried out in the laboratory electric furnace LAC-LH 30/13. Two tablets from each series were placed on a refractory fireclay plate at room temperature and the temperature increased in accordance with the predetermined programme.

Heating proceeded by a gradual temperature increase to target values of 850, 900, 950, and 1000 °C, which were reached after approximately 2 h (850 °C), 2.5 h (900 °C), 3 h (950 °C), and less than 3.5 h (1000 °C). After the target temperature was reached, the control program maintained the selected value for the prescribed calcination time according to the experimental plan, after which the furnace was switched off. The calcined clinkers were removed from the furnace after cooling to room temperature. From each pair of calcined tablets of a given type, one was used for subsequent leaching and analytical evaluation, while the other was archived as a reserve sample.

An appropriate structure and particle size of the calcination product are essential for achieving the most uniform possible contact between the solid phase and the leaching solution. Therefore, the effect of post-calcination clinker preparation on the efficiency of lithium transfer to solution was also evaluated. For comparison, two variants were prepared: a sample ground in a mortar and a sample subjected to one milling cycle. Based on the comparison of lithium transfer efficiency, a unified procedure for preparing the calcination product was selected for the subsequent experimental series.

Lithium transfer to solution was achieved by leaching the clinker, and the process efficiency was evaluated based on chemical analysis of the obtained filtrate. To ensure comparable conditions among individual experiments, the leaching procedure was standardized and carried out under defined temperature and stirring conditions.

For each experiment, 15 g of clinker was weighed, to which 150 g of deionized water preheated to 90 °C was added, corresponding to a clinker:water mass ratio of 1:10. The process was carried out for 30 min under continuous stirring (300 rpm) at a constant leaching-medium temperature of 90 °C, maintained by electric bottom heating controlled by a digital thermostat with a temperature probe placed directly in the stirred solution.

After completion of leaching, the solid and liquid phases were separated by vacuum filtration on a Büchner funnel using circular pieces of quantitative filter paper intended for fine-grained samples (particle retention approximately 2–3 μm).

For quantitative evaluation of the mass balance and transfer efficiency, chemical analyses were performed by ICP-MS. For each experimental sample, the lithium concentration was determined in the clinker, the leaching solution, and the insoluble residue after leaching.

The inherent variability of the overall experimental procedure on mass-balance recalculation, the fraction of lithium transferred to solution and the residual lithium content in the solid phase were determined. It was also possible to assess, in an indicative way, the balance of some other elements in the technological process, including accompanying alkali metals (especially rubidium). The main

evaluation criterion for comparing the effect of individual experiments was the efficiency of lithium transfer to solution.

Under identical conditions (950 °C, 60 min), five tablets of identical composition corresponding to the standard batch mixture with laboratory additives (lithium concentrate : $CaSO_4$: $Ca(OH)_2$ = 6 : 4.2 : 2) were calcined in parallel.

Leaching of the clinkers was carried out by the same procedure as in the previous experiments (15 g clinker charge; clinker-to-water ratio 1:10; temperature 90 °C; leaching time 30 min). The lithium transfer efficiency was evaluated by mass-balance recalculation based on lithium determination in the clinker and filtrate by ICP-MS.

The obtained data set was used to determine the mean transfer efficiency and the confidence interval, which serves as a quantitative indicator of the reproducibility of the experimental methodology. Terminological note: To evaluate the efficiency of the transfer of elements from one phase to another is used the term "transfer efficiency". For lithium as the central element in this paper, the alternative term "lithium recovery" is also used.

Table S10. Determination of the confidence interval of lithium transfer efficiency in parallel calcination–leaching experiments (mixture 1, n = 5).

Sample	Li in clinker (mg)	Li in filtrate (mg)	Li transfer (%)	Difference between the highest and lowest value	Number of measurements	Reliability coefficient Kn	Mean of the determined efficiencies	Confidence interval (95%)
1/V1	59.81	60.48	101.11					
1/V2	57.39	60.76	105.86					
1/V3	65.61	60.93	92.86					
1/V4	66.77	60.52	90.63					
1/V5	64.09	59.89	93.45	10.48	5	0.51	96.78	± 5.31

Note 1: The confidence interval was determined according to the relation $L_{D,H} = X \pm K_n \cdot R$, where X is the mean lithium transfer efficiency, R is the difference between the highest and lowest value, and Kn is a coefficient dependent on the number of measurements. For n = 5 and a significance level of 0.05, Kn = 0.507 was used. Note 2: Mass-balance values higher than 100% are caused by the analytical uncertainty of ICP-MS determination and by the accumulation of mass deviations in the individual sample-processing steps.

Table S11. Mass balance of selected elements (Ca, K, Li, Rb) between the clinker, filtrate, and insoluble residue after leaching — parallel samples 1/V1 to 1/V5.

1/V1

Element	In clinker charge (mg)	In filtrate (mg)	Transferred (%)	In insoluble residue (mg)	Residue (%)
Ca	2723.04	71.76	2.64	2814.73	103.37
K	424.71	165.22	38.90	326.34	76.84
Li	59.81	60.48	101.11	9.29	15.53
Rb	58.17	5.58	9.59	55.55	95.50

1/V2

Element	In clinker charge (mg)	In filtrate (mg)	Transferred (%)	In insoluble residue (mg)	Residue (%)
Ca	2641.24	77.95	2.95	2822.80	106.87
K	476.92	163.28	34.24	319.56	67.00
Li	57.39	60.76	105.86	8.90	15.51
Rb	55.80	5.66	10.14	55.54	99.54

1/V3

Element	In clinker charge (mg)	In filtrate (mg)	Transferred (%)	In insoluble residue (mg)	Residue (%)
Ca	3081.22	65.66	2.13	2802.62	90.96
K	484.00	166.74	34.45	333.03	68.81
Li	65.61	60.93	92.86	8.64	13.18
Rb	61.53	5.79	9.40	55.43	90.07

1/V4

Element	In clinker charge (mg)	In filtrate (mg)	Transferred (%)	In insoluble residue (mg)	Residue (%)
Ca	2961.79	67.77	2.29	2808.26	94.82
K	474.66	166.94	35.17	323.82	68.22
Li	66.77	60.52	90.65	8.85	13.25
Rb	63.70	5.71	8.96	55.93	87.80

1/V5

Element	In clinker charge (mg)	In filtrate (mg)	Transferred (%)	In insoluble residue (mg)	Residue (%)
Ca	2937.13	60.97	2.08	2740.89	93.32
K	517.69	165.10	31.89	330.13	63.77
Li	64.09	59.89	93.45	8.76	13.68
Rb	62.46	5.60	8.97	54.58	87.39

Note: The table presents the mass balance of selected elements between the clinker, filtrate, and insoluble residue after leaching. Values exceeding 100% are a consequence of ICP-MS analytical uncertainty and the accumulation of mass deviations during sample processing.

Chapter S2.3. Effect of concentrate particle size on lithium transfer efficiency

Three variants of lithium concentrate corresponding to different intensities of mechanical treatment were compared: unmilled concentrate, 1× milled, and 2× milled.

The particle-size variants of the concentrate were processed within reference batch mixture 1 with laboratory calcination additives. The mass ratios of the individual mixture components were kept constant in all cases (lithium concentrate : $CaSO_4$: $Ca(OH)_2$ = 6 : 4.2 : 2), and the only variable was the particle-size distribution of the lithium concentrate.

Table S12. Transfer of selected elements to solution at different degrees of mechanical treatment of the lithium concentrate (ZC) (15 g clinker charge; clinker-to-water ratio 1:10; temperature 90 °C; leaching time 30 min).

Element	unmilled ZC			1× milled ZC			2× milled ZC		
	in clinker (mg)	in filtrate (mg)	transferred (%)	in clinker (mg)	in filtrate (mg)	transferred (%)	in clinker (mg)	in filtrate (mg)	transferred (%)
Ca	2748.64	67.77	2.47	2798.33	62.81	2.24	2982.32	67.3	2.26
K	430.1	123.11	28.62	452.14	143.42	31.72	507.58	166.18	32.74
Li	60.57	49.49	81.71	63.5	57.86	91.13	60.74	59.88	98.58
Rb	58.24	5.84	10.02	61.72	5.14	8.34	59.51	5.6	9.4

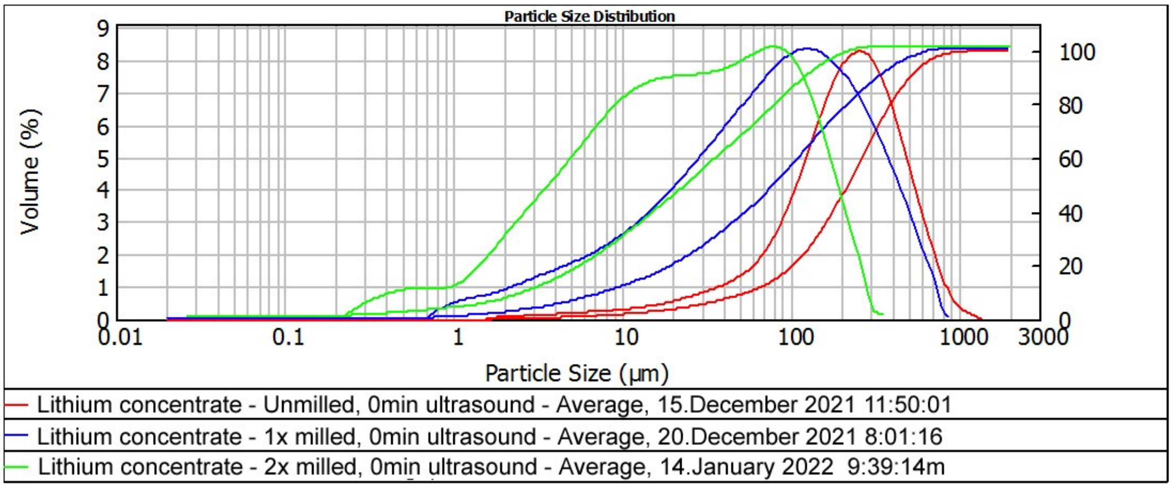

Figure S11. Particle-size distribution of the lithium concentrate for three particle-size variants.

Table S13. Particle-size distribution of the lithium concentrate for three particle-size variants.

Sample	Parameter	D(0.1)	D(0.5)	D(0.9)	D(4.3)
Unmilled	[µm]	55.09	218.40	510.13	257.63
1× milled	[µm]	8.45	80.86	335.70	132.07
2× milled	[µm]	2.54	22.37	119.89	44.27

Table S14. Mass-balance evaluation of selected elements after clinker leaching – unmilled lithium concentrate.

Element	In clinker charge (mg)	In filtrate (mg)	Transferred (%)	In insoluble residue (mg)	Residue (%)
Ca	2748.64	67.77	2.47	2823.56	102.73
K	430.10	123.11	28.62	340.90	79.26
Li	60.57	49.49	81.71	11.27	18.60
Rb	58.24	5.84	10.02	49.79	85.49

Note 1: The table presents the mass balance of selected elements between the clinker, filtrate, and insoluble residue after leaching. Leaching conditions: 15 g clinker charge, clinker:water ratio = 1:10, temperature 90 °C, time 30 min, stirring 300 rpm. Note 2: Mass-balance values higher than 100% are caused by the analytical uncertainty of ICP-MS determination and by the accumulation of mass deviations in the individual sample-processing steps.

Table S15. Mass-balance evaluation of elements after clinker leaching – 1× milled lithium concentrate.

Element	In clinker charge (mg)	In filtrate (mg)	Transferred (%)	In insoluble residue (mg)	Residue (%)
Ca	2798.33	62.81	2.24	2722.91	97.31
K	452.14	143.42	31.72	328.28	72.61
Li	63.50	57.86	91.13	4.92	7.75
Rb	61.72	5.14	8.34	53.70	87.00

Table S16. Mass-balance evaluation of elements after clinker leaching – 2× milled lithium concentrate.

Element	In clinker charge (mg)	In filtrate (mg)	Transferred (%)	In insoluble residue (mg)	Residue (%)
Ca	2982.32	67.30	2.26	2815.53	94.41
K	507.58	166.18	32.74	311.84	61.44
Li	60.74	59.88	98.58	3.10	5.11
Rb	59.51	5.60	9.40	53.39	89.72

Chapter S2.4. Effect of clinker particle size on lithium transfer to solution

The experiment verified, from a technological point of view, the effect of calcined clinker particle size on the leaching process.

From clinker calcined under identical conditions (950 °C, 60 min), a pair of samples differing in the degree of mechanical treatment was prepared: 1/D1 (unmilled, crushed in a mortar) and 1/D2 (1× milled, 300 rpm, corundum balls with a diameter of 3 cm, 10 min).

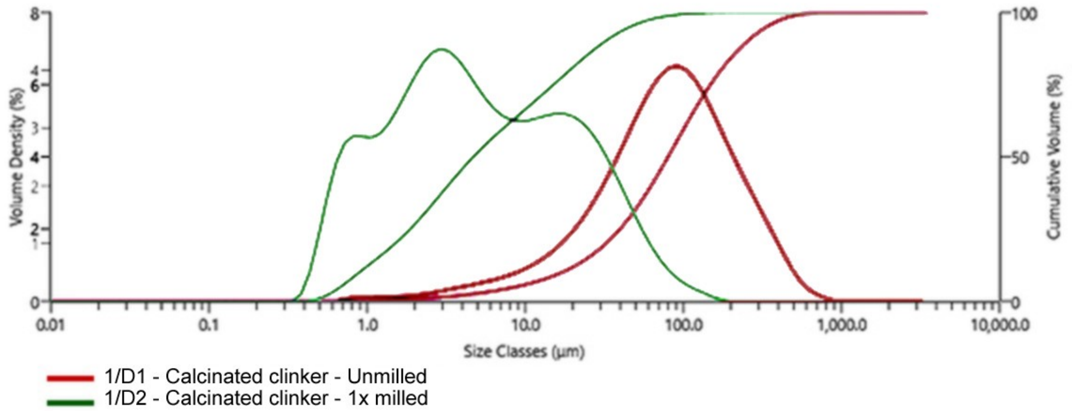

Figure S12. Particle-size distribution of calcined clinker for variants without subsequent milling (1/D1) and after a single milling step in a planetary mill (1/D2).

Table S17. Particle-size distribution of calcined clinker for variants without subsequent milling (1/D1) and after a single milling step in a planetary mill (1/D2).

Sample	Parameter	D(0.1)	D(0.5)	D(0.9)	D(4.3)
Unmilled	[µm]	17.0	80.0	254	113
1x milled	[µm]	0.89	4.69	32.0	11.9

Leaching was carried out under identical conditions for both samples, and the lithium transfer efficiency was determined by mass-balance recalculation based on the concentrations in the clinker and filtrate.

Table S18. Transfer of selected elements to solution as a function of mechanical treatment of the calcined clinker (6 g charge; clinker:water ratio = 1:10; temperature 90 °C; leaching time 30 min).

Element	1/D1 – crushed clinker			1/D2 – 1× milled clinker		
	in clinker (mg)	in filtrate (mg)	transferred (%)	in clinker (mg)	in filtrate (mg)	transferred (%)
Ca	1192.93	11.56	0.97	1192.93	14.36	1.20
K	203.03	65.91	32.46	203.03	63.06	31.06
Li	24.30	23.75	97.76	24.30	21.19	87.22
Rb	23.80	2.41	10.11	23.80	2.06	8.67

Table S19. Mass balance of selected elements (Ca, K, Li, Rb) between the clinker, filtrate, and insoluble residue after leaching — samples 1/D1 and 1/D2.

1/D1

Element	Clinker (mg)	Filtrate (mg)	Transferred (%)	In insoluble residue (mg)	Residue (%)
Ca	1192.93	11.56	0.97	1221.95	102.43
K	203.03	65.91	32.46	136.47	67.21
Li	24.30	23.75	97.76	1.10	4.54
Rb	23.80	2.41	10.11	22.91	96.23

1/D2

Element	Clinker (mg)	Filtrate (mg)	Transferred (%)	In insoluble residue (mg)	Residue (%)
Ca	1192.93	14.36	1.20	1223.03	102.52
K	203.03	63.06	31.06	128.79	63.43
Li	24.30	21.19	87.22	2.57	10.59
Rb	23.80	2.06	8.67	20.59	86.50

Note: The table presents the mass balance of selected elements between the clinker, filtrate, and insoluble residue after leaching. Values exceeding 100% are a consequence of ICP-MS analytical uncertainty and the accumulation of mass deviations during sample processing.

Chapter S2.5. Effect of calcination conditions on lithium transfer

The aim was to determine the combination of calcination temperature and time leading to maximum lithium extraction from the lithium concentrate for each batch mixture differing in the nature of the calcium-containing additives used.

Three batch mixtures were prepared: one representing a laboratory-defined reference system and two more technologically realistic variants using industrial raw materials.

Table S20. Mixing ratios and weighed amounts of the components used in the mixtures.

Reference Mixture	lithium concentrate 2× milled	Sulfate component: Synthetic ($CaSO_4 \cdot 2H_2O$) p.a.	$Ca(OH)_2$ p.a.
Mixing ratio (wt. parts)	6	4.2	2
20 g tablet	9.84 g	6.89 g	3.28 g
Natural gypsum Mixture	lithium concentrate 2× milled	Sulfate component: natural gypsum from the Kobeřice mine	Hydrated lime CL 90-S
Mixing ratio (wt. parts)	6	4.3	2
20 g tablet	9.76 g	6.99 g	3.25 g
FGD gypsum Mixture	lithium concentrate 2× milled	Sulfate component: FGD gypsum from power plant Ledvice	Hydrated lime CL 90-S
Mixing ratio (wt. parts)	6	3.6	2
20 g tablet	10.34 g	6.21 g	3.45 g

The tablets prepared by pressing were calcined in an electric furnace at temperatures of 850, 900, 950, and 1000 °C and calcination times of 15, 30, 60, and 90 min. In each individual experiment, all three compared mixtures were calcined simultaneously in the furnace.

Chapter S2.6. Elemental analysis of lithium concentrate and other calcination additives and products by ICP-MS

Procedure: The sample charge (0.1 ± 0.01 g) was digested in a mixture of 3 mL HF (38–40%, p.a.; Lach:ner), 1 mL HCl (35%, p.a.; Penta), and 1 mL HNO_3 (65%, p.a.; Lach:ner) using an Anton Paar Multiwave 5000 microwave digestion system.

The following digestion program was used: the temperature of the digestion mixture in all vessels was increased at approximately 10–15 °C/min to (185 ± 5) °C and maintained at this temperature for (20 ± 1) min. After cooling, the solutions containing the digested sample were transferred to 50 mL centrifuge tubes. To prevent etching of the glass parts of the ICP-MS by hydrofluoric acid, 20 mL of saturated boric acid was first added to each solution with the digested sample. All tubes were then filled with deionized water to a final volume of 50 mL and mixed.

By this method, the samples were completely digested. The solutions were then centrifuged (4,000 rpm, 10 min) and diluted tenfold with deionized water. The diluted solutions were analyzed using an Agilent 7900 ICP-MS instrument (instrument configuration: argon plasma torch, ion optics, ORS 4 collision/reaction cell (4th generation Octopole Reaction System), hyperbolic quadrupole, and orthogonal detector system).

QA/QC: Before the actual measurement, instrument tuning (adjustment) was performed (tuning solution: 1 ppb Li, Co, Y, Ce, Tl). During measurement, indium internal standard at a concentration of 100 ppb was continuously introduced. A five-point calibration series of 0.1, 0.5, 1, 5, and 10 mg/L was prepared from calibration standard Astasol AN9090MN containing a mixture of 26 elements, and four-point calibration series of 0.1, 0.5, 1, and 5 mg/L was prepared from CRM AN9097MN for phosphorus, CRM AN9098MN for antimony, and CRM AN9045(1N) for rubidium. Together with the samples, a digestion blank was prepared and measured in the same way (digestion acids without sample, whose trace concentration was subtracted from the result). Each sample was digested and analysed twice, and the reported results are always the mean concentration from these two digestions.

Chapter S3. Results of calcination experiments with various reaction mixtures

Calcination experiments were performed using procedures described above. Results are summarized in the following tables.

Table S21. Lithium recovery for reference mixture as a function of calcination temperature and time. (The recovery calculated from the lithium balance and expressed in %)

Temperature calcination	15 min	30 min	60 min	90 min
850 °C	0.77	0.38	0.66	1.26
900 °C	30.87	46.19	68.33	74.58
950 °C	75.46	89.56	95.46	85.86
1000 °C	83.13	84.32	83.45	82.01

Table S22. Lithium recovery for natural gypsum mixture as a function of calcination temperature and time.

Temperature of calcination	15 min	30 min	60 min	90 min
850 °C	0.86	0.82	1.27	4.88
900 °C	22.29	39.93	71.30	77.61
950 °C	72.14	86.66	85.22	93.74
1000 °C	83.20	85.84	82.77	83.06

Table S23. Lithium recovery for FGD gypsum mixture as a function of calcination temperature and time.

Temperature of calcination	15 min	30 min	60 min	90 min
850 °C	0.35	0.68	0.64	0.49
900 °C	1.07	1.32	18.86	35.13
950 °C	60.63	84.60	90.58	93.09
1000 °C	88.79	88.25	85.06	86.52

Chapter S4. Verification of reaction transformations by XRD analysis

The analyses were deliberately focused on selected representative samples corresponding to characteristic states of the system, without the need to analyze all experimental combinations of process parameters. The selection included both clinkers corresponding to the conditions of maximum lithium transfer efficiency and samples representing limiting temperature and time regimes that made it possible to follow the progressive decomposition of zinnwaldite and the development of new mineral phases.

The aim of the X-ray diffraction analysis was to verify the completeness of decomposition of the initial silicate structure of zinnwaldite and to identify the solid phases in which lithium is bound after calcination. At the same time, it was assessed whether the mineralogical composition of clinkers prepared from laboratory and real calcination raw materials differed significantly. The analysis was focused on qualitative identification of crystalline phases and verification of the course of reaction transformations. Quantitative determination of their proportions (e.g., by the Rietveld method) was not the aim of this work. In the first stage, the X-ray diffraction analysis was focused on clinkers corresponding to combinations of calcination temperature and time at which the individual mixtures reached maximum lithium transfer efficiency to solution as determined by ICP-MS mass-balance evaluation. These samples represent the reaction state of the system corresponding to technologically optimal calcination conditions.

Table S24. Identified crystalline phases of clinkers from reference mixture, natural gypsum mixture and FGD gypsum mixture at maximum lithium transfer efficiency (XRD).

Mixture	Calcination conditions	Identified phases	Formula / note
Reference mixture	950 °C / 60 min	Anhydrite	$CaSO_4$ (residual sulfate component)
		Lithium sulfate	Li_2SO_4
		Lithium potassium sulfate	$LiKSO_4$
		Silicon dioxide	SiO_2
		Cuspidin	$Ca_4Si_2O_7F_2$
Natural gypsum mixture	950 °C / 90 min	Anhydrite	$CaSO_4$ (residual sulfate component)
		Lithium sulfate	Li_2SO_4
		Lithium potassium sulfate	$LiKSO_4$
		Silicon dioxide	SiO_2
		Cuspidin	$Ca_4Si_2O_7F_2$
FGD gypsum mixture	950 °C / 90 min	Anhydrite	$CaSO_4$ (residual sulfate component)
		Lithium sulfate	Li_2SO_4
		Lithium potassium sulfate	$LiKSO_4$
		Silicon dioxide	SiO_2
		Cuspidin	$Ca_4Si_2O_7F_2$

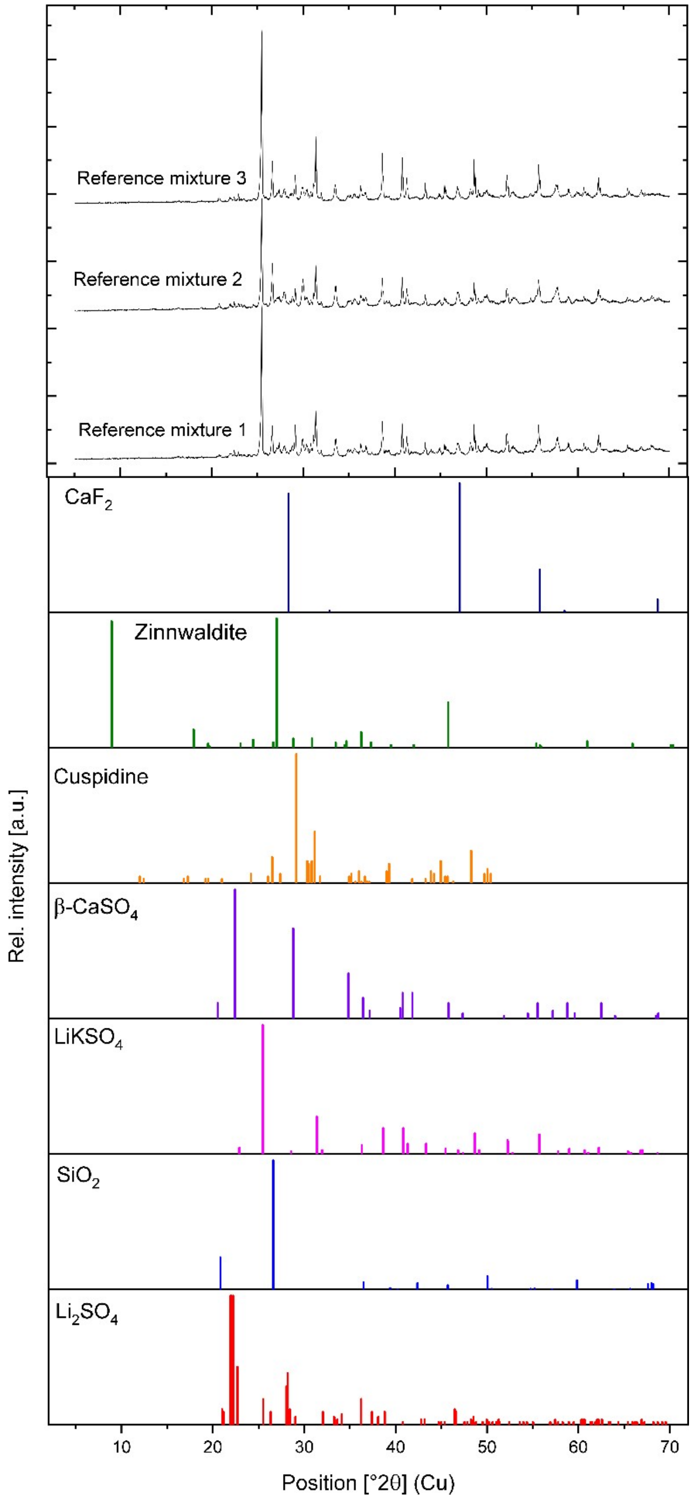

Figure S13. XRD diffraction pattern of clinker from reference mixture (950 °C / 60 min), mixture 2 (950 °C / 90 min) and mixture 3 (950 °C / 90 min).the X-ray diffraction analysis was focused on verifying the effect of calcination temperature on the course of reaction transformations in this mixture.

The X-ray diffraction focused on the course of transformations in the mixture containing the FGD gypsum - most promising variant for possible industrial applications.

It was examined whether a complete decomposition of zinnwaldite occurs at the investigated calcination temperatures and at the maximum investigated time of 90 min, and how the mineralogical composition of the clinker changes with increasing temperature. The results of crystalline-phase identification are summarized in the following table.

Table S25. Identified crystalline phases of clinkers from FGD gypsum mixture as a function of calcination temperature (calcination time 90 min, XRD).

Mixture	Calcination conditions	Identified phases	Formula / note
FGD gypsum mixture	850 °C / 90 min	zinnwaldite	$KAl(FeLi)(Si_3l)O_{10}F_2$
		Anhydritee	$CaSO_4$ (residual sulfate component)
		Silicon dioxide	SiO_2
		Calcium fluoride	CaF_2
FGD gypsum mixture	900 °C / 90 min	zinnwaldite	$KAl(FeLi)(Si_3l)O_{10}F_2$
		Anhydritee	$CaSO_4$ (residual sulfate component)
		Lithium sulfate	Li_2SO_4
		Lithium potassium sulfate	$LiKSO_4$
		Silicon dioxide	SiO_2
		Calcium fluoride	CaF_2
FGD gypsum mixture	950 °C / 90 min	Anhydritee	$CaSO_4$ (residual sulfate component)
		Lithium sulfate	Li_2SO_4
		Lithium potassium sulfate	$LiKSO_4$
		Silicon dioxide	SiO_2
		Cuspidin	$Ca_4Si_2O_7F_2$
FGD gypsum mixture	1000 °C / 90 min	Anhydritee	$CaSO_4$ (residual sulfate component)
		Lithium sulfate	Li_2SO_4
		Lithium potassium sulfate	$LiKSO_4$
		Silicon dioxide	SiO_2
		Cuspidin	$Ca_4Si_2O_7F_2$

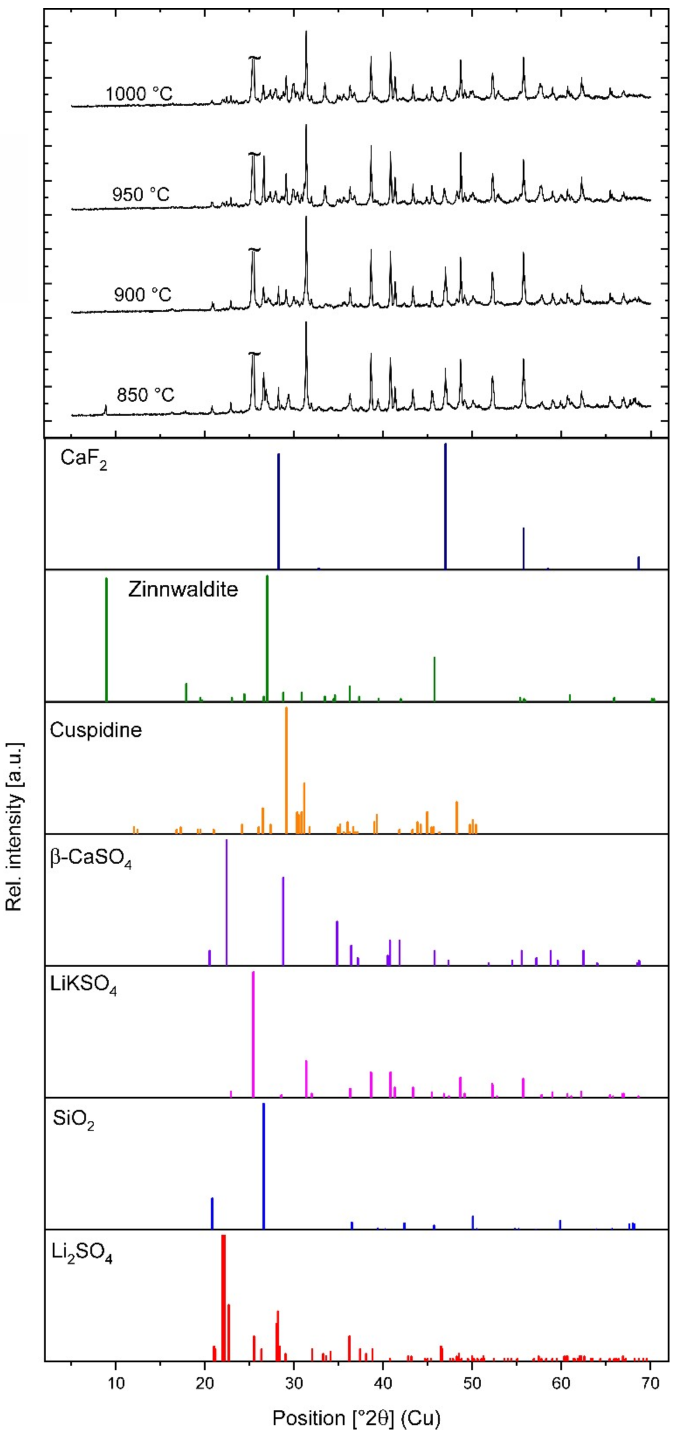

Figure S14. XRD diffraction pattern of clinker from FGD gypsum mixture at 850, 900. 950 and 1000 °C annealed for 90 min).

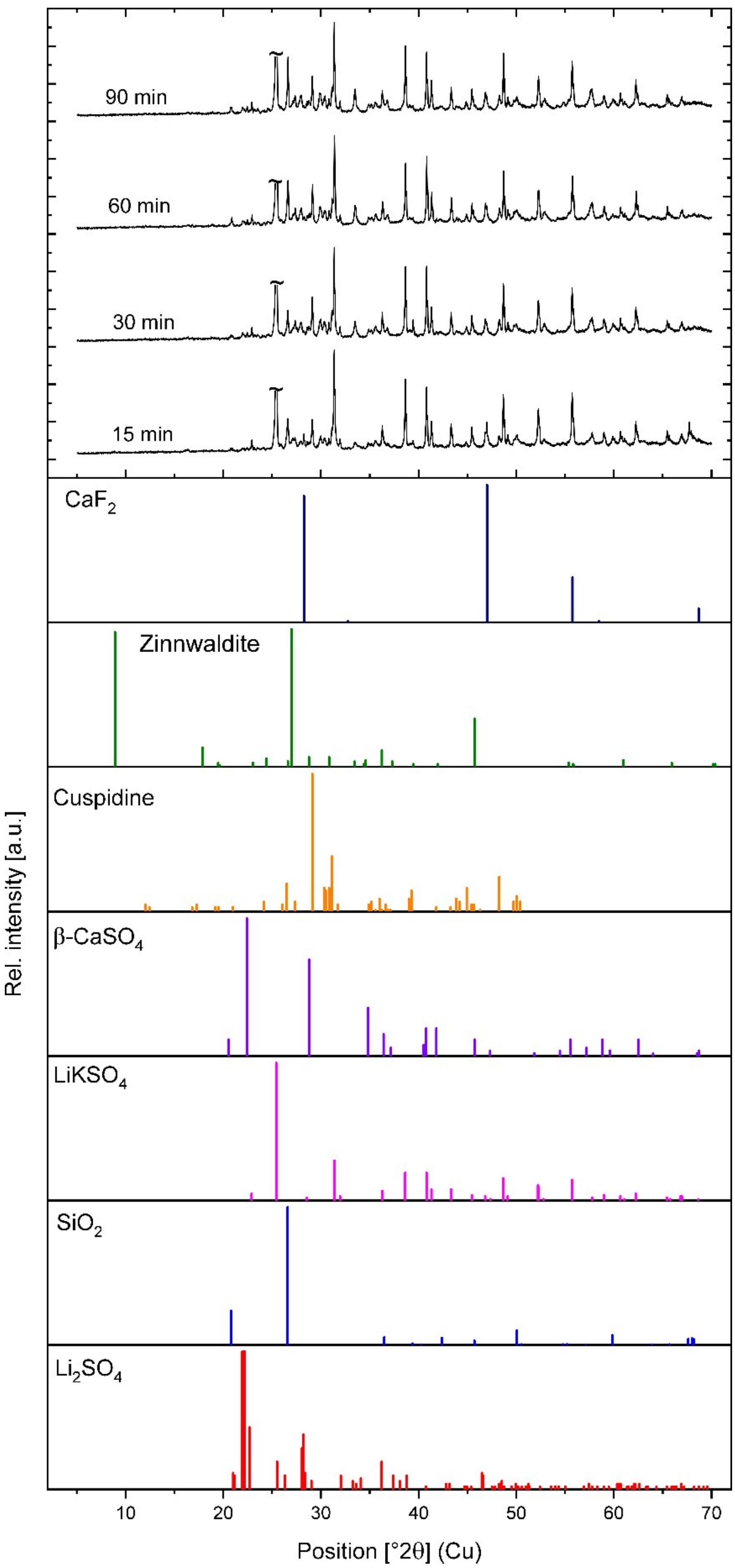

Figure S15. XRD diffraction pattern of clinker from FGD gypsum mixture at 950 °C for 15, 30, 60, and 90 min.

Chapter S5. Appendix. Bateman-type model for lithium recovery from zinnwaldite

Lithium extraction may be treated as a sequential solid-state activation process followed by aqueous leaching. Lithium initially bound in zinnwaldite, Lz, is converted during calcination into a transient leachable solid form, Ls (lithium sufates). The same intermediate may, however, be progressively lost by incorporation into refractory silicate, aluminosilicate or glassy phases, Lg. This gives a Bateman-type response: the amount of leachable lithium first increases as Ls is formed and subsequently decreases as Ls is consumed by immobilization.

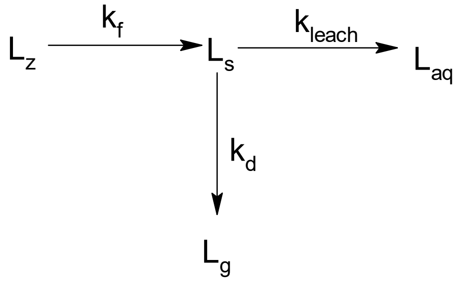

Here, k_f is the apparent rate constant for formation of the leachable lithium-bearing phase during calcination, kd is the apparent rate constant for its thermal deactivation/immobilization, and kleach is the rate constant for transfer of the remaining leachable lithium into the aqueous phase during the subsequent leaching step. For the calcination stage, the material balance is:

$$dLz/dt = -kf\, Lz$$
$$dLs/dt = kf\, Lz - kd\, Ls$$
$$dLg/dt = kd\, Ls$$

With Lz(0) = L0 and Ls(0) = Lg(0) = 0, the transient amount of leachable lithium is given by the Bateman expression:

$$\frac{Ls(t)}{L0} = \left[\frac{kd - kf}{kf}\right]\left[exp(- kf\ t) - exp(- kd\ t)\ \right]$$

The experimentally observed lithium recovery after calcination time t and a standardized leaching procedure can therefore be written as:

$$R(t) = \eta \cdot \left[\frac{kd - kf}{kf}\right][\exp(- kf\ t) - \exp(- kd\ t)]$$

where η is the leaching efficiency for the transient leachable phase. If leaching is fast and nearly quantitative for Ls, η approaches unity.

The time-dependent recovery data were evaluated using a formation–deactivation model based on a Bateman-type expression. In this approach, the measured lithium recovery is treated as the observable consequence of two consecutive or competing processes: the formation of leachable lithium-bearing

phases and their subsequent loss of leachability due to high-temperature transformation, incorporation into less reactive matrices, or sintering-related effects.

For most datasets, the model provided a satisfactory description of the increasing part of the recovery curve. In cases where no decrease in recovery was observed within the experimental time window, the deactivation constant was very small and the model effectively reduced to a pseudo-first-order growth-type behaviour. The selected examples in Fig. S16 illustrate the main kinetic regimes observed in this study. At 900 °C, the reference and natural gypsum mixtures show a gradual increase in lithium recovery and can be described by the formation-dominated part of the Bateman-type model. For FGD gypsum at 950 °C, the conversion is substantially faster and high recoveries are reached within short calcination times, again consistent with rapid formation of leachable lithium phases. At 1000 °C, the FGD gypsum mixture exhibits high initial recovery followed by a slight decrease with time. This behaviour can also be represented within the same model framework, although the apparent rate constant associated with the decreasing branch is two to three orders of magnitude lower than the rate constant for the formation step. This indicates that the high-temperature loss of leachability is comparatively slow but becomes relevant during prolonged calcination.

The FGD gypsum mixture at 900 °C represents a distinct case. Its recovery curve is sigmoidal rather than pseudo-first-order or Bateman-like in the initial region. This suggests the presence of an induction period preceding the efficient formation of leachable lithium species. The induction behaviour may reflect a required solid-state reorganisation, delayed generation of reactive sulfate/calcium phases, diffusion limitations, or the need to reach a threshold degree of transformation before lithium becomes accessible to leaching. Thus, the FGD 900 °C dataset indicates that this temperature is close to the lower boundary of the effective processing window for the FGD-based mixture.

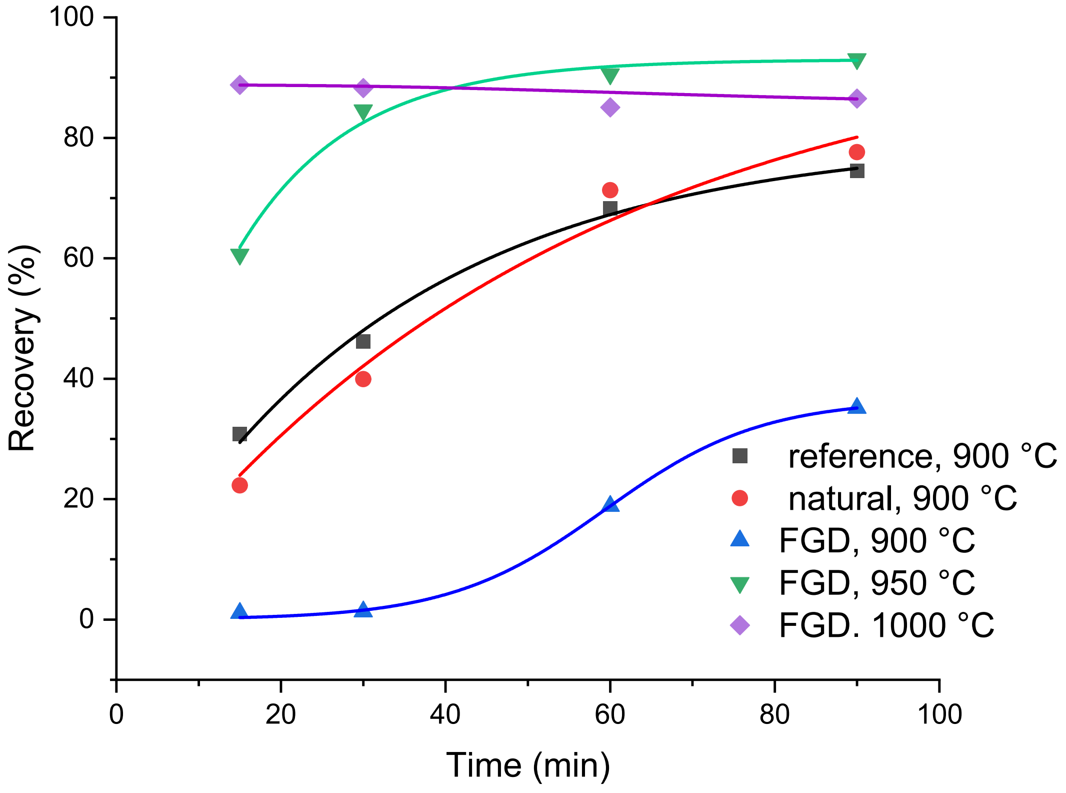

Figure S16. Dependencies of the lithium recovery on the time of calcination for selected mixtures and temperatures.

Table S26. Model suitability and model parameters for selected systems

Reaction mixture/temperature	Model suitability	Model parameters
FGD/900 °C	Sigmoidal, not consistent with Bateman	
FGD/950 °C	Pseudo-1st order (Eq.1)	$k_f = 0.073 \pm 0.004$; $R_\infty = 93.01 \pm 1.42$<br>$R^2 = 0,9836$
FGD/1000 °C	Decreasing; consistent with Bateman	$k_d \approx 10^{-4}$
natural/900 °C	Pseudo-1st order (Eq.1)	$k_f = 0.019 \pm 0.005$; $R_\infty = 98.79 \pm 14.02$<br>$R^2 = 0,9714$
reference/900 °C	Pseudo-1st order (Eq.1)	$k_f = 0.031 \pm 0.003$; $R_\infty = 80.07 \pm 2.73$<br>$R^2 = 0,9917$
